# Dietary diversity indices *v*. dietary guideline-based indices and their associations with non-communicable diseases, overweight and energy intake: evidence from China

**DOI:** 10.1017/S1368980022000556

**Published:** 2023-05

**Authors:** Jiajun Zhou, Sirimaporn Leepromrath, De Zhou

**Affiliations:** 1 College of Economics and Management, China Center for Food Security Studies, Nanjing Agricultural University, No. 1, Weigang, Xuanwu District, Nanjing 210095, China; 2 Agricultural Production and Resource Economics, Technical University of Munich, Freising, Germany

**Keywords:** Diet quality measurement, Dietary diversity, Non-communicable diseases, China

## Abstract

**Objectives:**

To evaluate various diet quality indices and to estimate their associations with major non-communicable diseases (NCD) (i.e. diabetes mellitus (DM) and myocardial infarction (MI)) and risk for overweight (OW).

**Design:**

Four dietary diversity indices (namely, count index (Count), dietary diversity score index, berry index (BI) and entropy index (EI)) and three Chinese dietary guideline-based indices (namely, China healthy diet index, Chinese food pagoda score and diet quality divergence index) were employed to evaluate Chinese diet quality. DM, MI and OW were used as diet-related health indicators. Logit regressions were employed to unveil the associations between diet quality indices and NCD and risk for OW. The relationships between diet quality indices and daily energy intakes were checked with ordinary least squares linear regressions.

**Setting:**

Four recent waves (2004, 2006, 2009, 2011) of longitudinal individual data from China Health and Nutrition Survey.

**Participants:**

Chinese adults (aged 18–64 years) from twelve provinces were included in the analysis (*n* 30 350).

**Results:**

Count, BI, and EI were positively associated with higher OW risk and daily energy intakes. As dietary guideline-based indices got better, people were exposed to lower DM and OW risks and got lower daily energy intakes. Finally, dietary guideline-based indices properly revealed the expected relationships that high-quality diets would reduce NCD and risk for OW, while high diversity indices were usually correlated with over-nutrition and high risks.

**Conclusions:**

Increasing diversity of the diet does not necessarily improve the nutrition and health. Dietary guideline-based indices are more robust than dietary diversity indices; thus, they should be highly recommended when evaluating diet quality.

China has been experiencing a remarkable nutrition transition with the rapid economic growth during the past decades^(1,2)^. The Chinese diet patterns have been gradually switching from the traditional diet, which was dominated by cereals and vegetables, towards the patterns associated with high-fat and energy density foods^(2,3)^. The nutrition transition has strong impacts on the diet quality and health. As one emerging economy, China encounters both over-nutrition^(2,4,5)^ and increasing non-communicable diseases (NCD)^(6,7)^. Currently, China has the largest overweight (OW) and diabetes mellitus (DM) population in the world^(6,8)^. The prevalence of OW among adults dramatically increased from 23·2 to 55·8 % in 1989–2011^(9)^, and the all-age prevalence of DM rose from 3·7 % in 1990 to 6·6 % in 2016^(8)^, with an increase of over 3000 patients/d^(10)^. In addition, myocardial infarction (MI) remains one of the most important causes of death in China with one million annual deaths^(11)^. MI mortality was estimated to increase 5·6 folds from 1987 to 2014^(12)^. NCD (i.e. DM and MI) and risk for OW (hereafter denoted as NCD and risk for OW) all together lead big health challenges in China^(6,7,13)^.

There are many studies focused on nutrition transition in China and some of them indicated that high-quality diet would significantly reduce diet-related NCD (e.g. DM^(14)^ and MI^(15)^) and risk for OW^(16)^. However, given the various foods and diet habits, it is worthy to note the complexity of diet quality and its measurements^(3,17)^. Generally, there are several ways to measure diet quality, which could roughly fall into two approaches: dietary diversity indices and dietary guideline-based indices. In the previous literature, dietary diversity indices, such as count index (Count), dietary diversity score (DDS), berry index (BI) and entropy index (EI), which mainly record the number of food items/groups consumed, and even the food distribution (namely, the varied amounts of food consumed in grams over the survey period), were frequently employed to measure diet quality^(5,18,19)^. Those measurements are easy to apply but have limited power to reveal the nutrition and health implications^(20)^. Meanwhile, most dietary guideline-based indices are composed based on dietary patterns and dietary guidelines for specific population published by health institutions, including Mediterranean diet scale^(21)^ for Mediterranean people, health eating index^(22)^ and diet quality index^(23)^ for Americans, China healthy diet index (CHDI)^(24)^, Chinese food pagoda score (CFPS)^(1)^, diet quality divergence (DQD) index^(17)^ and Chinese healthy eating index^(25)^ for Chinese. Dietary guideline-based indexes generally take account of the information on food distribution, food attributes and dietary habits. However, the cut-off weights for different food items/groups were usually designed by researchers according to their studies. One big concern associated with such kind of diet quality measurements is the subjective nature in the composing process and that would undermine the precision of the evaluation of diet quality to varying extent^(17)^.

All those diet quality indices which are proposed based on the various information on food attributes, diet habits and diet guidelines may pose their pros and cons and their nutrition and health implications may not be consistent with each other. For instance, some studies showed that there was a rising diet quality in past years in China^(1,5,17)^, while some other studies suggested that Chinese diet quality decreased in the past decade^(3)^. In addition, it is well known that income growth has crucial impacts on food consumption and diet quality improvement, especially in developing countries^(26)^. While some other studies reveal that the increasing income does not necessarily improve the diet quality^(3,27)^. Given the complexity of food consumption and diet quality, systematic studies on the various diet quality indices and their differences in diet evaluation and health implications are necessary, but still scant.

Therefore, this study will empirically adopt an unified framework to scrutinise the dynamics of Chinese diet quality and evaluate commonly used diet quality measurements, including four dietary diversity measurements (i.e. Count, DDS, BI and EI) and three dietary guideline-based indices (i.e. CHDI, CFPS and DQD), and their associations with major NCD, risk for OW and daily energy intakes with the use of most recent four waves (2004, 2006, 2009 and 2011) data from China Health and Nutrition Survey (CHNS). The specific purposes of the present study are as follows: (1) to evaluate the Chinese diet quality in 2004–2011 with the use of seven commonly used diet quality indices; (2) to further improve the research on diet quality measurements, especially on the differences between dietary diversity indices and guideline-based indices with the unified evaluation framework; and (3) to explore implications for the diet quality evaluation and NCD alleviation in China.

## Materials and methods

### Study participants

Four waves (2004, 2006, 2009 and 2011) of CHNS data were employed in this study. The CHNS was jointly implemented by the Carolina Population Center at the University of North Carolina at Chapel Hill and the National Institute for Nutrition and Health under Chinese Center for Disease Control and Prevention. It was an ongoing tracking survey of approximately 4000 families and 12 000 individuals per wave covering both urban and rural regions in nine provinces (Guangxi, Guizhou, Henan, Heilongjiang, Hubei, Hunan, Jiangsu, Liaoning and Shandong) in China before 2011, and three more autonomous cities (Beijing, Chongqing and Shanghai) after 2011^(28,29)^. A multistage, random cluster process was used to draw the samples in each province (autonomous city). Specifically, counties in each province were stratified by income (low, middle and high), and a weighted sampling scheme was used to randomly select four counties in each province. In addition, the provincial capital and a lower income city were selected when feasible. Villages and townships within the counties, urban and suburban neighbourhoods within the cities were selected randomly. More detailed information about CHNS can be found elsewhere^(28,29)^.

**Fig. 1:**
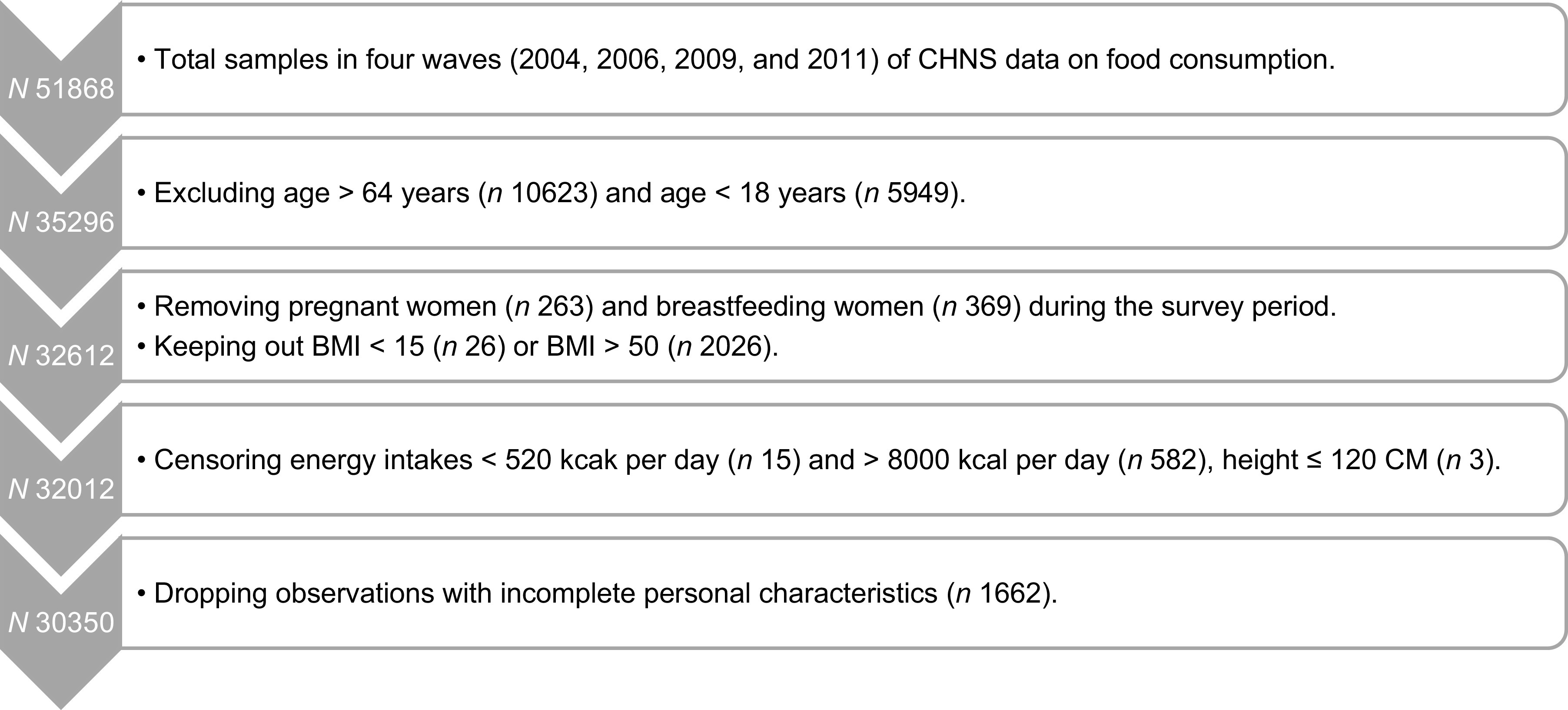
The process of sample selection

Figure 1 shows the samples selection process in the present study. There were 51 868 observed participants in total in the four waves of CHNS data on food consumption. Respondents older than 64 or younger than 18 were removed due to the variation of diet recommendations across different age groups in Chinese Dietary Guidelines (CDG) 2016^(30,31)^. Besides, pregnant women and breast-feeding women were excluded due to the special diet recommendations for these female populations in CDG 2016^(2,30)^. Observations with extremely abnormal BMI(< 15 or BMI > 50) were dropped to get representative samples^(2,17)^. Furthermore, following previous literature, samples with implausible energy intakes, including those lower than 520 kcal/d (minimum energy required for survival) and greater than 8000 kcal/d (about 4 times as much mean energy intakes), were further pruned away^(1,17)^. In addition, individuals who were below 120 cm in height (generally considered to be patients with human short stature) were excluded^(32)^. Observations with incomplete personal characteristics were also removed. Finally, 30 350 observations from the four waves of CHNS unbalanced longitudinal data were employed in the present study.

According to Table 1, we got 7025 (23·2 %), 6846 (22·6 %), 7229 (23·8 %) and 9250 (30·5 %) individual observations in 2004, 2006, 2009 and 2011, respectively. Meanwhile, there were 3525, 3520, 3613 and 4673 household observations in 2004, 2006, 2009 and 2011, respectively. Over the period of 2004–2011, only 2526 individual respondents in 1879 households were surveyed in all four waves in this study. The distribution of household size across years can be found in Fig. A.1 in appendix A. The present study pooled the data in 2004–2011 and carried out the analysis with dummy variables for different surveys.

**Table 1:** The distribution of observations in the dataset in 2004–2011

Wave	Individual observations				Household observations			
	*n*	%	Tracked		*n*	%	Tracked	
2004	7025	23·2	4810	2526	3525	23·0	2891	1879
2006	6846	22·6			3520	23·0		
			4467				2696	
2009	7229	23·8			3613	23·6		
			4942				2902	
2011	9250	30·5			4673	30·5		
Total	30 350				15 331			

### Assessment of food consumption

The nutrition survey was implemented in three consecutive days. Detailed daily food consumption data (in g) of each family member were collected in CHNS using a 24-h recall method, including all food items participants consumed at home and away from home. Following the previous studies, the individual food recall data for three consecutive days were summed up and then divided by three to obtain the individual average daily intake in this study^(3,17)^. All dietary data were recorded by trained interviewers through face-to-face structured interviews with use of food pictures and models, including ingredient codes (based on China Food Composition Table)^(33,34)^ and amounts of all consumed food items in breakfast, lunch and dinner^(35)^. All interviewers were trained by nutritionists or professionally engaged in nutrition work in their own counties or participated in other related surveys.

Individuals (aged ≥ 18 years) were asked to recall all food intakes during the last 24 h every survey day^(36)^. Respondents were prompted about shared dishes. To reduce the recall bias, other household members were encouraged to provide additional information to estimate the precise amount of food intakes. Finally, food consumed by individuals at home, restaurants, canteens and away from home was systematically recorded. Furthermore, changes of food inventory in the household in each day were also collected and individual food intakes based on 24-h recall method were compared with average daily food intakes calculated from the household food inventory survey to control the quality of nutrition data. When significant differences were found, the individuals were revisited correspondingly and their food intakes were double-checked^(28)^. More detailed information about the nutrition survey in CHNS can be found in the other literature^(28,35)^.

### Measurement of diet quality

As mentioned above, diet quality is usually measured by dietary diversity indices and dietary guideline-based indices^(17,20)^. In general, poor people usually consume limited cheap food products, while rich people have more options and can choose more diverse food products due to a larger budget^(37)^. A greater diversity often means an increase of consumer welfare and improved diet as they can enjoy more different foods^(38)^. Therefore, dietary diversity indices can be useful measures of diet quality, and they could be generally classified into two groups: count indices which only record the number of food items/groups, and distribution indices which take account of both the number of food items/groups and the distribution of the various amounts of food consumed over the survey period^(19)^. Two count indices (i.e. Count and DDS) and two distribution indices (i.e. BI and EI) were employed in this study. Moreover, three dietary guideline-based indices (i.e. CHDI, CFPS and DQD) were adopted.

The Count Index (Count) was proposed by Kant^(39)^, which is the total number of food items (based on food codes from China Food Composition Table 2002–2004^(33,34)^) consumed by the individuals with the range of. A higher Count indicates a more diverse food consumption and high-quality diet.

The DDS counts the number of food groups daily consumed by individuals^(40)^. In the previous literature^(19)^, based on similarities in nutrient composition and dietary function, food items are aggregated into six food groups to calculate DDS as follows: (1) cereal and potatoes, (2) fruits, (3) vegetables, (4) aquatic products/meat/poultry, (5) legumes/nuts/eggs and (6) milk and milk products. According to the study of Wang *et al*.^(19)^, eggs are grouped with legumes and nuts, and the latter are mainly made up of tofu and milk in kind which are important protein sources for Chinese. Strictly following such food grouping method, we also estimated DDS by counting the number of consumed food groups mentioned above. In addition, following the previous studies^(19)^, food groups consumed less than 25 g/d were excluded, except for dairy products with the minimum amount of 10 g/d due to the relatively low consumption of dairy products in China. Therefore, DDS ranges from 0 to 6, and the bigger the DDS, the better the dietary diversity.

Moreover, not only the number of food products but also the exact amounts of consumed foods have important influence on diet quality due to the existence of different marginal utilities of various foods for consumers. The distribution indices thus take the number and distribution into consideration, and among them, BI and EI were adopted in the present study. Following the previous literature^(18,19)^, BI is defined as a function of the food share ( 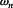 ) and computed as follows:
(1)

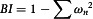

where 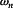 denotes the share of 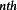 food item in the total amount of food (in g) consumed by the participant over the survey period. BI ranges 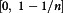 .

EI is also calculated with the food share ( 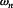 ) but it put greater weight on small food share values^(19)^. Thus, EI is particularly sensitive to the minor items in the food basket and ranges 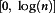 ^(18)^:
(2)

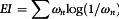

Both BI and EI take account of the variety of food items ( 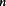 ) and the distribution of the amounts of consumed foods in grams. Given the food variety ( 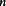 ), BI and EI become bigger when the values of food share ( 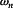 ) are close to each other and get maximised when food shares of different food items are precisely equal that also indicate a balanced food consumption and high-quality diet.

Taking the representative of measures into consideration, three dietary guideline-based indices were employed in the present study. All those indices are composed based on CDG 2016 for general adults, including CHDI^(24)^, CFPS^(1)^ and DQD index^(17)^. CDG 2016 was jointly composed by the Chinese Center for Disease Control and Prevention (CDC), National Health and Family Planning Commission of the People’s Republic of China and the Chinese Nutrition Society^(30)^. The Chinese food pagoda (CFP) 2016 succinctly shows the daily recommendations of eight food categories for general adults (aged 18–64 years) (Table 2)^(31)^: (1) cereal and potatoes; (2) fruits; (3) vegetables; (4) eggs; (5) aquatic products (fish, shellfish and mollusk); (6) meat and poultry; (7) legumes and nuts; and (8) milk and milk products. Following the previous studies^(17,36)^, salt and oil were excluded in the present study due to their imprecise individual intakes measured with the changes of inventory in CHNS.

**Table 2:** Recommendations in Chinese food pagoda (CFP) 2016

Category	Food group	Recommended intake (g/d)
(1)	Cereal and potatoes	250–400
(2)	Fruits	200–350
(3)	Vegetables	300–500
(4)	Eggs	40–50
(5)	Aquatic products	40–75
(6)	Meat and poultry	40–75
(7)	Legumes and nuts	25–35
(8)	Milk and milk products	≥ 300

Source: Chinese dietary guidelines 2016, China food composition table 2002–2004.

Following the suggestion of previous studies^(5,24)^, the CHDI is composed according to the consumed food groups and scores (Table 3). For simplicity, the original nine major food categories are combined into seven broad groups (cereal and potatoes, vegetables, fruits, dairy products, legumes and nuts, meat and poultry and eggs, aquatic products) based on similarities in nutrients and dietary functions^(5)^. Types of food in CHDI refer to the daily average number of food items ( 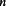 ) participant consumed over the survey period. The scores for eight components are summed up to obtain CHDI. Finally, CHDI ranges from 0 to 70 and a higher CHDI indicates a better diet quality.

**Table 3:** China healthy diet index (CHDI) scoring standards

Component	Maximum score	Maximum standard	Minimum score	Minimum standard
Original component				
Types of food	10	≥ 12 types	0	≤ 5 types
Refined grains	5	≥ 100 g/1000 kcal	0	0
Whole grain, dry bean and tuber	5	≥ 40 g/1000 kcal	0	0
Total vegetables	5	≥ 180 g/1000 kcal	0	0
Dark green and orange vegetables	5	≥ 90 g/1000 kcal	0	0
Fruits	10	≥ 110 g/1000 kcal	0	0
Milk and milk products	10	≥ 100 g/1000 kcal	0	0
Legumes and nuts	10	≥ 10 g/1000 kcal	0	0
Meat, poultry and eggs	5	≥ 50 g/1000 kcal	0	0
Aquatic products	5	≥ 30 g/1000 kcal	0	0
Present component				
Types of food	10	≥ 12 types	0	≤ 5 types
Cereal and potatoes	10	≥ 140 g/1000 kcal	0	0
Vegetables	10	≥ 270 g/1000 kcal	0	0
Fruits	10	≥ 110 g/1000 kcal	0	0
Milk and milk products	10	≥ 100 g/1000 kcal	0	0
Legumes and nuts	10	≥ 10 g/1000 kcal	0	0
Meat, poultry and eggs	5	≥ 50 g/1000 kcal	0	0
Aquatic products	5	≥ 30 g/1000 kcal	0	0

Types of food in CHDI refer to the daily average number of food items ( 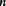 ) consumed by the participant over the survey period.

To keep consistent with CFP 2016 and the original food grouping method for CFPS^(1)^, all food items consumed by individuals are summed up into eight food groups when estimating CFPS (Table 4). Under-consumption or over-consumption takes place when the individual dietary consumption is lower than the lower bound or higher than the upper bound of corresponding recommendation level in CFP 2016. Following the scoring method of CFPS in previous literature^(1,41)^, each food group gets score ‘1’ if the consumption settles in the recommended interval, ‘0·5’ if the consumption locates in 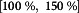 of the upper bound or 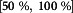 of lower bound, and “0” otherwise. Finally, CFPS is obtained by summing up all the scores for eight food groups and ranges from 0 to 8, and the higher the CFPS index, the better the diet quality.

**Table 4:** Chinese food pagoda score (CFPS) across various energy levels

Food group	1600 kcal	1800 kcal	2000 kcal	2200 kcal	2400 kcal	2600 kcal	2800 kcal
Cereal and potatoes (g)							
Score as ‘1’	175–225	200–250	225–275	250–300	275–325	325–375	350–400
Score as ‘0·5’	88–175	100–200	113–225	125–250	138–275	163–325	175–350
Score as ‘0·5’	225–338	250–375	275–413	300–450	325–488	375–563	400–600
Vegetables (g)							
Score as ‘1’	≥ 300	≥ 400	≥ 450	≥ 450	≥ 500	≥ 500	≥ 500
Score as ‘0·5’	150–300	200–400	225–450	225–450	250–500	250–500	250–500
Fruits (g)							
Score as ‘1’	≥ 200	≥ 200	≥ 300	≥ 300	≥ 350	≥ 350	≥ 400
Score as ‘0·5’	100–200	100–200	150–300	150–300	175–350	175–350	200–400
Meat and poultry (g)							
Score as ‘1’	15–65	25–75	25–75	50–100	50–100	50–100	75–125
Score as ‘0·5’	8–15	13–25	13–25	25–50	25–50	25–50	38–75
Score as ‘0·5’	65–98	75–113	75–113	100–150	100–150	100–150	125–188
Eggs (g)*							
Score as ‘1’	40–50						
Score as ‘0·5’	20–40						
Score as ‘0·5’	50–75						
Aquatic products (g)							
Score as ‘1’	≥ 40	≥ 50	≥ 50	≥ 75	≥ 75	≥ 75	≥ 100
Score as ‘0·5’	20–40	25–50	25–50	38–75	38–75	38–75	50–100
Milk and milk products (g)*							
Score as ‘1’	≥ 300						
Score as ‘0·5’	150–300						
Legumes and nuts (g)							
Score as ‘1’	15–25	15–25	15–25	25–35	25–35	25–35	25–35
Score as ‘0·5’	8–15	8–15	8–15	13–25	13–25	13–25	13–25
Score as ‘0·5’	25–38	25–38	25–38	35–53	35–53	35–53	35–53

* Means the same recommended cut-off intervals for all energy levels.

The energy level is the upper bound for each interval. For instance, individuals with energy intakes lower than or equal to 1600 kcal are classified into the group ‘1600’.

The DQD is composed with the cumulative absolute divergence between the diets and recommendations in CFP 2016 (Table 2)^(17)^.
(3)

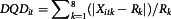

where 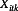 is the average daily intake of food category 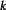 for individual 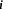 in year 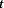 , 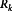 is the vector of daily recommendations in CFP 2016. Given 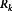 is the interval for some food categories, when 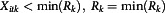 , and when 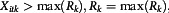 and when 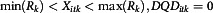 . DQD ranges [0, +∞], and one smaller DQD indicates a better diet quality and vice versa. Particularly, when DQD gets close to 0, the diet pattern is fully consistent with recommendations from CFP 2016.

### Measurement of non-communicable diseases and risk for overweight

Due to the lack of checkup data (e.g. blood biochemistry test, electrocardiogram examination), respondents’ self-report on NCD was employed to identify DM or myocardial infraction (MI). DM was recorded in CHNS by asking each participant directly ‘has a doctor ever told you that you suffer from diabetes mellitus’. The record for DM was one if the respondent answered yes, and zero otherwise. However, CHNS did not distinguish type 1 diabetes mellitus and type 2 diabetes mellitus. Thus, DM indicated two types of diabetes altogether in this study. Following the same way of DM, each participant was asked to answer ‘has a doctor ever given you the diagnosis of myocardial infarction’, and the record for MI was one if the answer was yes and zero otherwise.

Regarding risk for OW, BMI, namely the weight (kg) divided by square of the height (m), is widely used to measure OW for adults^(2,16,17)^. Thus, BMI was employed to measure the body shape and OW in this study. In CHNS, anthropometric data (height in cm) and weight in kg) were measured directly by well-trained health workers based on a standard protocol recommended by the WHO^(35)^. According to the recommendations from working group on obesity in China, there were two categorical BMI levels, namely OW (BMI ≥ 24 kg/m^2^) and non-OW (BMI < 24 kg/m^2^) in this study^(2)^.

### Measurement of other covariates

Some important information, such as income, demographics (e.g. age, gender), labour force participation, activity data for each individual, was also collected in CHNS^(28)^. In this study, the natural logarithm of per capita annual net income was employed as the measure of income, which was derived from the annual total household income divided by the number of the family members and deflated by consumer price index at 2015 prices^(17)^. The highest level of education each participant had attained was adopted as individual education level (1 = no school completed; 2 = primary school; 3 = lower middle school; 4 = upper middle school; 5 = vocational degree; 6 = undergraduate or higher degrees)^(1,3)^.

Daily exercise time (e.g. running, football, gymnastics, etc.) and sedentary time (e.g. watching TV, playing video games, etc.) were recorded in CHNS by asking the participant how much time (minutes) he/she generally spent in a typical day from Monday to Friday, and how much time they took on weekends (Saturday and Sunday). The exercise and sedentary time the respondents spent in these two periods were summed up and divided by two to measure individual daily exercise and sedentary activity within 1 week (Monday to Sunday), respectively^(17)^.

Labour intensity level was recorded according to the occupation in CHNS (1 = light physical activity, working in a sitting or standing position like office worker, salesperson, laboratory technician; 2 = moderate physical activity, e.g. student, driver, electrician; 3 = heavy physical activity, such as farmer, steel worker, loader, miner, stonecutter)^(1)^.

Drinking was measured with the frequency of alcohol drinks according to the response of participant (1 = no drinking; 2 = very low frequency, no more than once a month; 3 = low frequency, once or twice a month; 4 = medium frequency, once or twice a week; 5 = high frequency, 3–4 times a week; 6 = very high frequency, almost every day)^(17)^.

The household size, namely total number of family members within household, was also adopted^(1,17)^. To control the influential factors at community level, urbanisation level was employed^(41)^, which was measured by a multidimensional urbanisation index, including twelve factors in total such as population density, economic environment, transportation infrastructure and communications^(42)^.

### Methodology and model

Firstly, the dynamics of diet quality indices from 2004 to 2011 were reported by figures. Pairwise comparisons of means were adopted to compare the changes (2004, 2006, 2009 and 2011) of diet quality indices using Tukey’s adjustment in computing *P*-values. To explore the differences of the mean diet quality indices between different groups (i.e. DM *v*. non-DM, MI *v*. non-MI and OW *v*. non-OW), mean-comparison tests adjusted for gender were conducted. Furthermore, binary Logit regressions were adopted to estimate the associations between diet quality indices and NCD as well as risk for OW. The regression is based on the cumulative logistic probability function and specified as follows:
(4)

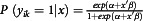

where 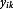 is a binary variable, 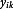 = 1 denotes individual 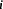 has NCD or risk for OW 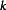 (i.e. DM or MI or OW) and 0 otherwise; 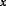 is a vector of 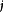 explanatory variables which could be either discrete or continuous, including personal characteristics (e.g. diet quality, age), household characteristics (e.g. household size), community level factors (e.g. urbanisation) and dummy variables for different year. 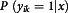 is the probability that individual 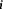 gets NCD or risk for OW 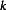 given the variables 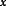 . Both 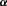 and 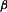 are parameters needed to be estimated. After estimating Logit regression model, the marginal effect of explanatory variable 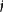 on the predicted probability of having NCD or risk for OW 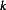 is given by partial derivation of 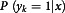 on 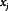 , setting all explanatory variables to their means. To explore the associations between diet quality indices and average daily energy intakes, ordinary least squares (OLS) linear regressions were conducted.

It should be noted that samples in this study were unbalanced longitudinal data with some observations from the same participants and family members from the same households. Diet quality indices for these respondents were correlated. Thus, the individual cluster effects and household cluster effects were controlled in Logit regression models and OLS models^(1)^.

All statistical tests were two-tailed tests in this study, and *P* < 0·05 was considered as statistically significant. Data analysis was performed in software package of STATA/MP 16.0.

## Results

### Descriptive analysis

Table 5 presents the descriptive statistics of diet quality indices and the covariates of the samples in this study. Males and females took up 47·7 and 52·3 %, respectively. The mean DDS, BI, EI, CHDI and CFPS of females were higher than that of males, while males generally had larger Count and DQD (divergence from dietary guidelines) than females. The prevalence of DM, MI and OW in males was higher than that in females. In general, male participants had higher education level than females. Besides, the labour intensity level of males was stronger than that of females. In addition, males drank more frequently than females. Meanwhile, the average number of cigarettes consumed by males was about twenty-nine times that of females. The mean daily energy intakes of males were generally higher than that of females.

**Table 5:** Characteristics of participants

Variables	Male (*n* 14 466)			Female (*n* 15 884)		
	*n*	Mean	sd *	*n*	Mean	sd *
Diet quality measurements						
Count	14 466	14·4	5·8	15 884	14·1	5·8
DDS	14 466	4·0	0·9	15 884	4·0	1·0
BI	14 466	0·8	0·1	15 884	0·8	0·1
EI	14 466	2·2	0·4	15 884	2·2	0·4
CHDI	14 466	55·9	5·1	15 884	56·1	5·2
CFPS	14 466	2·0	1·0	15 884	2·1	1·0
DQD	14 466	5·6	2·4	15 884	5·0	2·1
Health outcomes						
	%	%	%	%	%	%
Person with OW	5956	41·2		6293	39·6	
Person without OW	8510	58·8		9591	60·4	
Person with DM	306	2·1		282	1·8	
Person without DM	14 160	97·9		15 602	98·2	
Person with MI	57	0·4		60	0·4	
Person without MI	14 409	99·6		15 824	99·6	
Socio-economic variables						
Age (year)	14 466	44·8	11·9	15 884	45·2	11·5
Education						
	%	%		%	%	
No school completed	1179	8·2		3467	21·8	
Primary school	2586	17·9		3164	19·9	
Lower middle school	5702	39·4		5046	31·8	
Upper middle school	2509	17·3		2006	12·6	
Vocational degree	1157	8·0		1118	7·0	
Undergraduate degree or higher	1333	9·2		1083	6·8	
Income (ln (yuan/year/capita))	14 466	9·0	1·1	15 884	9·0	1·1
Sedentary (h)	14 466	3·1	2·4	15 884	2·8	2·2
Exercise (min)	14 466	0·2	0·6	15 884	0·1	0·5
Labour intensity†						
	%	%	%	%	%	%
Light	5946	41·1		8550	53·8 %	
Moderate	2962	20·5		2131	13·4 %	
Heavy	5558	38·4		5203	32·8 %	
Drinking‡						
No drinking	5400	37·3		14 336	90·3	
Very low	745	5·2		525	3·3	
Low	1734	12·0		439	2·8	
Medium	2429	16·8		322	2·0	
High	1291	8·9		101	0·6	
Very high	2867	19·8		161	1·0	
Smoking§	14 466	9·7	11·0	15 884	0·3	2·3
Energy intake (kcal)‖	14 466	2340	699	15 884	1963	600
Household size (persons)	14 466	3·7	1·5	15 884	3·7	1·5
Urbanisation¶	14 466	66·6	19·9	15 884	66·8	20·1
	%	%		%	%	
2004	3384	23·4		3641	22·9	
2006	3235	22·4		3611	22·7	
2009	3461	23·9		3768	23·7	
2011	4386	30·3		4864	30·6	

* sd is the acronym of sd.

† Labour intensity levels: 1 = light physical activity, working in a sitting or standing position (e.g. office work, watch smith, counter salesperson, lab technician); 2 = moderate physical activity (e.g. driver, electrician); and 3 = heavy physical activity (e.g. farmer, athlete, dancer, steel worker, lumber worker, mason).

‡ Drinking: 1 = no drinking; 2 = very low frequency, no more than once a month; 3 = low frequency, once or twice a month; 4 = medium frequency, once or twice a week; 5 = high frequency, 3–4 times a week; 6 = very high frequency, almost every day.

§ Smoking: the amount of cigarettes/d.

‖ Energy intake: the individual average daily energy intake constructed by CHNS.

¶ Defined by a multidimensional 12-component urbanisation index, including the population density, physical, social, cultural and economic environment.

### The dynamics of diet quality for Chinese adults between 2004 and 2011

**Fig. 2:**
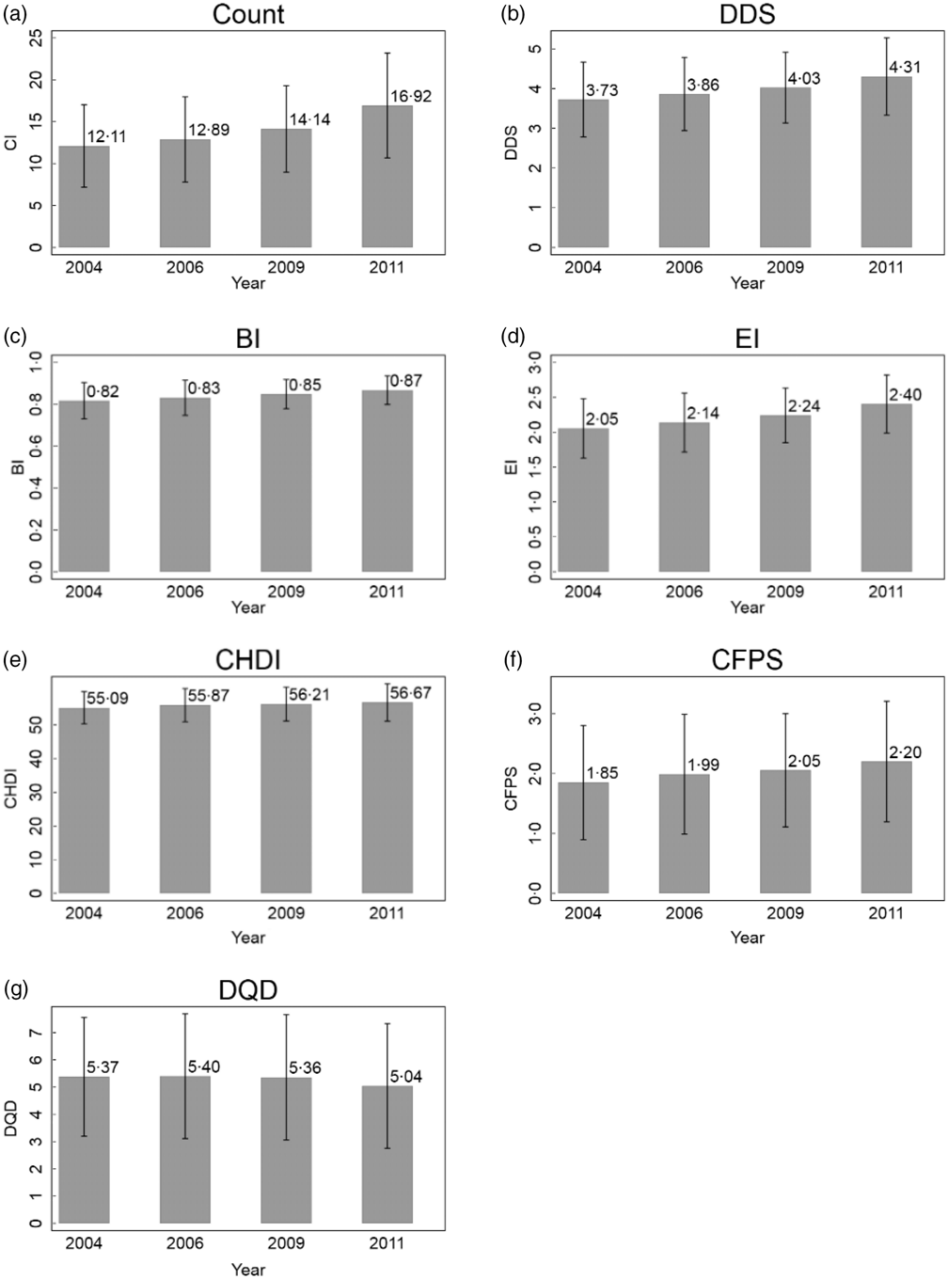
Dynamics of diet quality indices between 2004 and 2011. The bar refers to the mean diet quality indices, and the solid black line above the bar refers to mean ± sd

Figure 2 illustrates the values of Count, DDS, BI, EI, CFPS and CHDI continuously rose over 2004–2011, meanwhile the DQD which indicates the dietary divergence from the CFP 2016 generally declined.

Pairwise comparisons of Chinese diet quality indices across different waves are reported in Table 6. The values of Count, DDS, BI, EI, CHDI and CFPS increased over time, and all changes were statistically significant at 1 %. For example, value of Count significantly increased by 0·78 during the period of 2004–2006, rose by 1·25 during the period of 2006–2009. Declining DQD was observed over the time period (except for the period of 2004–2006).

**Table 6:** Comparisons of Chinese diet quality between each wave of surveys in China Health and Nutrition Survey

	2004 *v*. 2006		2004 *v*. 2009		2004 *v*. 2011		2006 *v*. 2009		2006 *v*. 2011		2009 *v*. 2011	
	Mean	SE	Mean	SE	Mean	SE	Mean	SE	Mean	SE	Mean	SE
Count	0·78	0·09	2·03	0·09	4·81	0·09	1·25	0·09	4·03	0·09	2·79	0·09
*P*	<0·01		<0·01		<0·01		<0·01		<0·01		<0·01	
DDS	0·14	0·02	0·30	0·02	0·58	0·01	0·16	0·02	0·44	0·01	0·28	0·01
*P*	<0·01		<0·01		<0·01		<0·01		<0·01		<0·01	
BI	0·01	<0·01	0·03	<0·01	0·05	<0·01	0·02	<0·01	0·04	<0·01	0·02	<0·01
*P*	<0·01		<0·01		<0·01		<0·01		<0·01		<0·01	
EI	0·08	0·01	0·19	0·01	0·35	0·01	0·10	0·01	0·27	0·01	0·16	0·01
*P*	<0·01		<0·01		<0·01		<0·01		<0·01		<0·01	
CHDI	0·78	0·09	1·12	0·09	1·58	0·08	0·34	0·09	0·80	0·08	0·46	0·08
*P*	<0·01		<0·01		<0·01		<0·01		<0·01		<0·01	
CFPS	0·14	0·02	0·21	0·02	0·35	0·02	0·07	0·02	0·21	0·02	0·15	0·02
*P*	<0·01		<0·01		<0·01		<0·01		<0·01		<0·01	
DQD	0·03	0·04	−0·02	0·04	−0·33	0·04	−0·05	0·04	−0·36	0·04	−0·31	0·04
*P*	0·90		0·96		<0·01		0·63		<0·01		<0·01	

Differences in means of diet quality indices across waves are reported.

For instance, Count value increased by 0 78 over the period of 2004–2006.

Standard errors (se) are provided in parentheses.

*P* values are computed through Tukey’s adjustment.

### The changes of non-communicable diseases prevalence and risk for overweight between 2004 and 2011

**Fig. 3:**
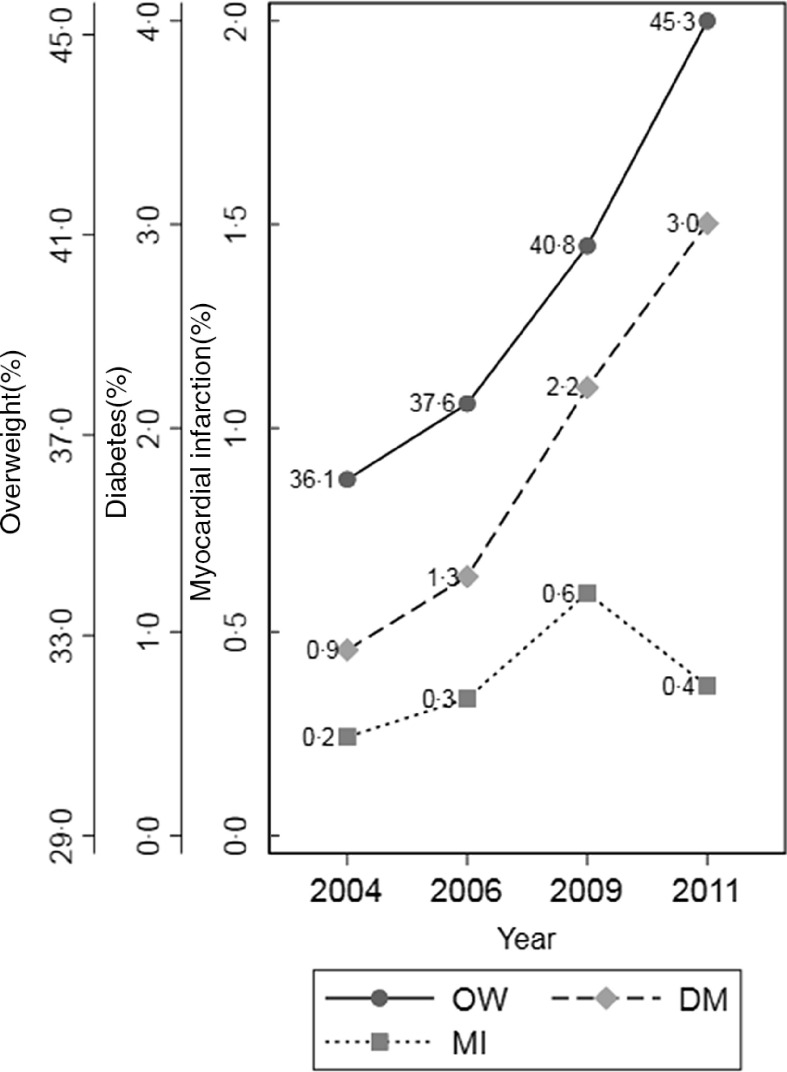
Changes in the prevalence of NCD for general Chinese adults according to CHNS: 2004–2011. OW, DM and MI are the acronyms of overweight, diabetes mellitus and myocardial infarction, respectively. Overweight is defined as BMI ≥ 24 kg/m^2^

Figure 3 illustrates the percentages of NCD and OW risk groups generally rose from 2004 to 2011. Specifically, the OW prevalence continuously grew from 36·1 to 45·3 %. The prevalence of DM rose from 0·9 to 3·0 %. The percentage of MI generally increased from 0·2 to 0·6 % during 2004–2009 and then decreased to 0·4 % in 2011.

### Diet quality status across different subpopulations

Table 7 illustrates the differences of diet quality indices between NCD and risk for OW group and their counterparts, which were tested by two samples *t*-test and adjusted for gender. Male participants with OW had a larger Count, DDS, BI, EI, CHDI, CFPS and DQD than non-OW male group (all *P* < 0·05). Meanwhile, women with OW tended to have a larger Count, DDS, BI, EI and DQD but lower CHDI and CFPS than their non-OW counterparts. Comparisons between DM group and non-DM group showed that participants with DM generally had a larger Count, DDS, BI, EI, CHDI, CFPS and DQD than their non-DM counterparts. In addition, the results also revealed that participants with MI tended to get larger Count, DDS, BI, EI, CHDI, CFPS and DQD than their non-MI counterparts (except for Count for female between MI group and non-MI group).

**Table 7:** Diet quality indices across subpopulations

	Count		DDS		BI		EI		CHDI		CFPS		DQD	
	Mean	sd	Mean	sd	Mean	sd	Mean	sd	Mean	sd	Mean	sd	Mean	sd
Male														
OW	15·32	5·81	4·10	0·91	0·85	0·08	2·30	0·42	56·03	5·28	2·03	0·96	5·62	2·55
Non-OW	13·70	5·64	3·91	0·92	0·83	0·08	2·16	0·43	55·78	4·98	1·92	0·95	5·54	2·29
Diff	1·62		0·20		0·02		0·13		0·26		0·11		0·08	
*P*	<0·01		<0·01		<0·01		<0·01		<0·01		<0·01		0·05	
DM	16·78	5·93	4·31	0·89	0·87	0·08	2·43	0·41	56·63	5·85	2·20	0·94	5·71	2·81
Non-DM	14·31	5·75	3·98	0·92	0·84	0·08	2·21	0·43	55·87	5·09	1·96	0·96	5·57	2·39
Diff	2·46		0·33		0·03		0·21		0·76		0·24		0·14	
*P*	<0·01		<0·01		<0·01		<0·01		0·01		<0·01		0·31	
MI	15·37	5·68	4·05	1·08	0·87	0·05	2·36	0·37	56·54	4·94	2·11	1·13	5·86	3·81
Non-MI	14·36	5·77	3·99	0·92	0·84	0·08	2·22	0·43	55·88	5·11	1·96	0·96	5·57	2·39
Diff	1·01		0·07		0·03		0·15		0·66		0·14		0·29	
*P*	0·19		0·60		<0·01		0·01		0·33		0·27		0·36	
Female														
OW	14·19	5·56	4·02	0·98	0·85	0·07	2·24	0·42	56·10	5·23	2·08	1·02	5·07	2·19
Non-OW	14·08	5·91	4·02	1·00	0·84	0·08	2·22	0·45	56·17	5·17	2·12	1·00	4·97	2·06
Diff	0·11		0·01		0·01		0·03		−0·07		−0·07		0·10	
*P*	0·25		0·74		<0·01		<0·01		0·43		0·01		<0·01	
DM	15·64	5·98	4·23	1·02	0·87	0·07	2·37	0·43	56·77	4·79	2·29	1·01	5·08	2·16
Non-DM	14·09	5·76	4·02	0·99	0·84	0·08	2·23	0·44	56·13	5·20	2·10	1·01	5·01	2·11
Diff	1·55		0·22		0·02		0·14		0·64		0·19		0·07	
*P*	<0·01		<0·01		<0·01		<0·01		0·04		<0·01		0·59	
MI	13·92	4·88	4·15	0·82	0·85	0·07	2·26	0·38	57·17	5·27	2·27	1·15	5·50	3·03
Non-MI	14·12	5·77	4·02	0·99	0·85	0·08	2·23	0·44	56·14	5·20	2·10	1·01	5·01	2·11
Diff	−0·21		0·13		0·01		0·03		1·03		0·17		0·49	
*P*	0·78		0·31		0·34		0·61		0·13		0·21		0·07	

OW, DM and MI are the acronyms of overweight, diabetes mellitus and myocardial infarction, respectively.

### Associations between diet quality indices and non-communicable diseases as well as risk for overweight

Table 8 reports the associations between diet quality indices and NCD as well as risk for OW based on binary Logit regressions with the control of demographic characteristics and regional variables using individual cluster effects and household cluster effects, respectively (referring to Tables A1–A4 in appendix A for detailed results). As all the diet quality indices were not binary variables, the average marginal effects were calculated given all the covariates valued at their averages. In addition, the individual posterior probabilities of NCD and risk for OW were predicted based on the estimated Logit models and independent variables. When we set up posterior probability of 0·5 as the switch point^(43)^, there were nearly 60 % of samples which were correctly classified into OW and non-OW groups; meanwhile, more than 98 % of samples could be properly classified for DM group *v*. non-DM group and MI *v*. non-MI groups. Thus, it is reasonable to employ Logit models to investigate the associations between diet quality indices and NCD as well as risk for OW.

**Table 8:** Average marginal effects of diet quality indices on non-communicable diseases and risk for overweight from Logit regressions with the use of individual/household cluster effect (*n* 30 350)

	Overweight		Diabetes mellitus		Myocardial infraction	
	Individual	Household	Individual	Household	Individual	Household
Count**	2·7‡	2·7‡	−3·5‖	−3·5‖	−1·8‖	−1·8‖
95 % CI	1·4‡, 4·1‡	1·3‡, 4·1‡	−3·6§, 2·9§	−3·6§, 2·9§	−1·8§, 1·5§	−1·9§, 1·5§
*P*	<0·01	<0·01	0·83	0·83	0·83	0·83
%	61·1	61·1	98·1	98·1	99·6	99·6
DDS**	2·0‡	2·0‡	−1·4‡	−1·4‡	−1·4§	−1·4§
95 % CI	−5·2‡, 9·2‡	−5·6‡, 9·6‡	−3·2‡, 4·6§	−3·2‡, 4·8§	−9·9§, 7·1§	−1·0‡, 7·3§
*P*	0·59	0·61	0·14	0·15	0·75	0·76
%	61·1	61·1	98·1	98·1	99·6	99·6
BI††	0·3	0·3	8·1‡	8·1‡	1·2†	1·2†
95 % CI	0·2, 0·4	0·2, 0·4	−2·6†, 4·2†	−2·6†, 4·2†	−1·4‡, 2·5†	−1·5‡, 2·5†
*P*	<0·01	<0·01	0·64	0·64	0·08	0·08
%	61·1	61·1	98·1	98·1	99·6	99·6
EI††	5·7†	5·7†	1·8‡	1·8‡	1·1‡	1·1‡
95 % CI	3·9†, 7·4†	3·7†, 7·6†	−3·4‡, 7·0‡	−3·4‡, 7·0‡	−1·0‡, 3·2‡	−1·1‡, 3·3‡
*P*	<0·01	<0·01	0·51	0·51	0·31	0·33
%	61·2	61·2	98·1	98·1	99·6	99·6
CHDI‡‡	−1·2‡	−1·2‡	−5·1‖	−5·1‖	7·5‖	7·5‖
95 % CI	−2·3‡, −3·5‖	−2·4‡, 2·2‖	−3·4§, 2·3§	−3·3§, 2·3§	−5·8‖, 2·1§	−5·7‖, 2·1‡
*P*	0·04	0·05	0·73	0·72	0·27	0·27
%	61·1	61·1	98·1	98·1	99·6	99·6
CFPS‡‡	−1·3†	−1·3†	−1·1‡	−1·1‡	9·7‖	9·7‖
95 % CI	−1·9†, −6·2‡	−1·9†, −5·9‡	−2·8‡, 5·3§	−2·8‡, 5·3§	−7·2§, 9·1§	−7·2§, 9·2§
*P*	<0·01	<0·01	0·18	0·18	0·81	0·82
%	61·1	61·1	98·1	98·1	99·6	99·6
DQD§§	5·1‡	5·1‡	7·7§	7·7§	2·8§	2·8§
95 % CI	2·6‡, 7·6‡	2·4‡, 7·7‡	2·3§, 1·3‡	2·2§, 1·3‡	−2·5‖, 5·8§	−2·6‖, 5·8§
*P*	<0·01	<0·01	0·01	0·01	0·07	0·07
%	61·1	61·1	98·1	98·1	99·6	99·6

*Statistically significant at *P* < 0 05.

† The coefficient is displayed in scientific notation format: coefficient × 10^−2^.

‡ Scientific notation format: coefficient × 10^−3^.

§ Scientific notation format: coefficient × 10^−4^.

‖ Scientific notation format: coefficient × 10^−5^.

**,††,‡‡,§§Denote the full estimated results for specified diet quality indices could be found in Tables A1–A4 in appendix A, respectively.

Associations are investigated by binary Logit regressions using individual/household cluster effects with the control for gender, age, education, household net income per capita in logarithm, daily sedentary activity time, daily exercise time, labour intensity level, frequency of drinking alcohol, household size, urbanisation index and dummy variables for years.

Overweight is defined as BMI ≥ 24.

Diabetes mellitus includes both type 1 and type 2 diabetes mellitus. (%) is the percentage of samples correctly classified.

According to the results, the Count value was positively associated with the risk for OW with average marginal effect of 0·3 % (*P* < 0·01). Even though DDS had positive correlation with risk for OW, negative relationships with DM and MI, all those relationships were not statistically significant. With the average marginal effect of 30 % (*P* < 0·01), BI was positively associated with the risk for OW. The Logit regressions of OW risk on EI indicated that risk for OW increased as EI became larger with the average marginal effect of 5·7 % (*P* < 0·01).

When it comes to dietary guideline-based indices, a higher CHDI was significantly associated with lower risk for OW (*P* < 0·05). Moreover, the results indicated a higher CFPS was negatively correlated with risk for OW, with the average marginal effects of −1·3 % (*P* < 0·01). Finally, a larger DQD, namely poorer diet quality, significantly associated with DM, MI and risk for OW, with the marginal effects of 0·5 % (*P* < 0·01), 0·1 % (*P* < 0·01) and 0·03 % (*P* < 0·1), respectively.

**Fig. 4:**
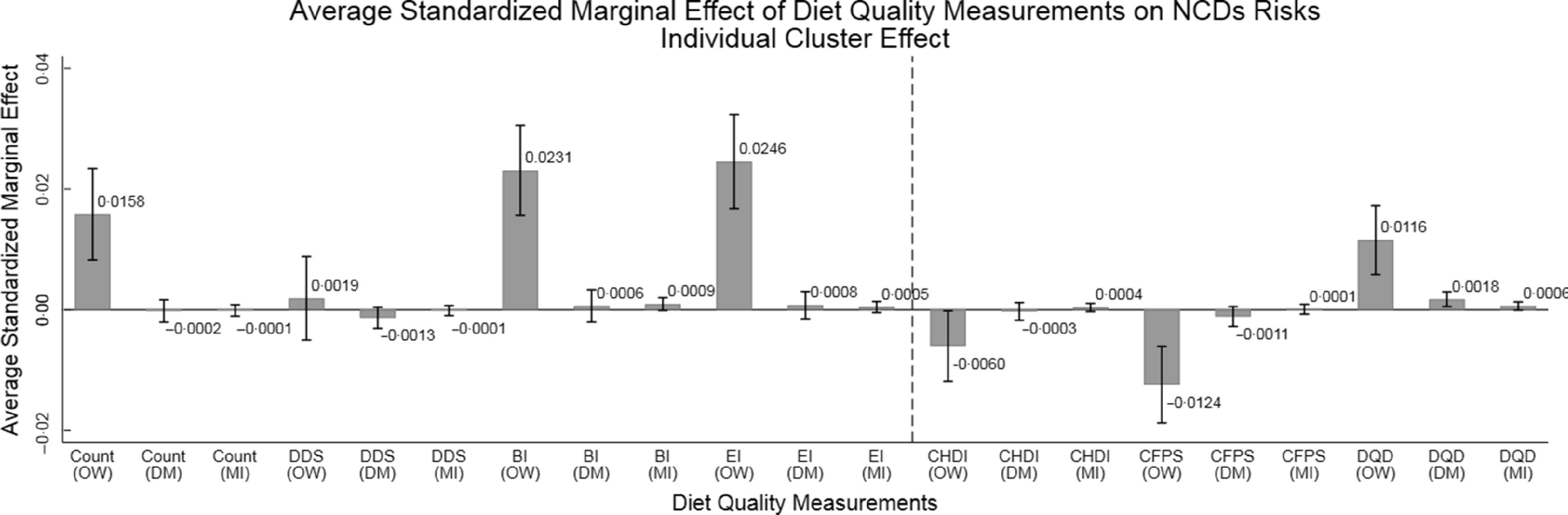
Average standardised marginal effects of diet quality indices on NCD risks from Logit regressions using individual cluster effect (*n* 30 350). OW, DM and MI are acronyms of overweight, diabetes mellitus and myocardial infarction, respectively. Overweight is defined as BMI ≥ 24 kg/m^2^. DM includes both type 1 and type 2 diabetes mellitus. The bar and the number above the bar refer to the mean standardised marginal effects, and the solid black short line above the bar refers to mean ± 95 % CI

**Fig. 5:**
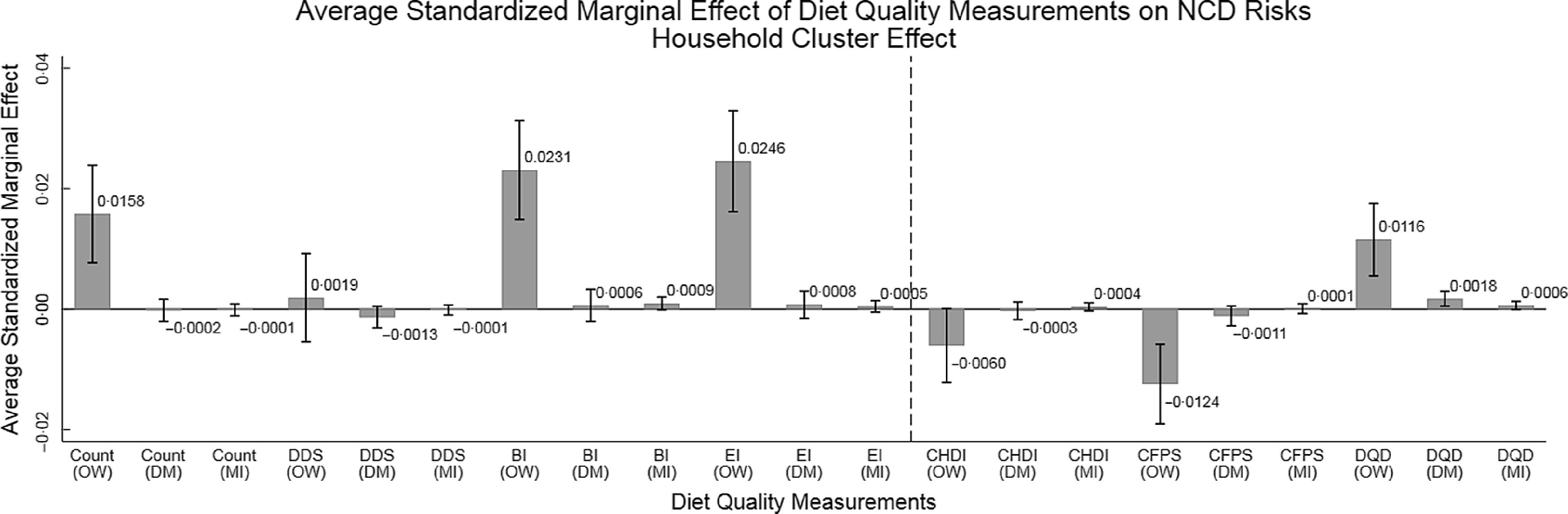
Average standardised marginal effects of diet quality indices on NCD risks from Logit regressions using household cluster effect (*n* 30 350). OW, DM and MI are acronyms of overweight, diabetes mellitus and myocardial infarction, respectively. Overweight is defined as BMI ≥ 24 kg/m^2^. DM includes both type 1 and type 2 diabetes mellitus. The bar and the number above the bar refer to the mean standardised marginal effects, and the solid black short line above the bar refers to mean ± 95 % CI

Furthermore, since the scales of various indices were different, to make all estimations more comparable, the present study also employed the standardised diet quality indices in Logit regressions. Figures 4 and 5 report the average standardised marginal effects of indices on NCD and risk for OW (namely, the associations between 1 SD increase of diet quality indices and the changes of the NCD and risk for OW) using individual cluster effects and household cluster effects, respectively.

### Associations between diet quality indices and daily energy intakes

Table 9 reports the associations between diet quality indices and average daily energy intakes from OLS linear regressions with individual cluster effects and household cluster effects. The models also took account of the demographic characteristics and regional variables. The results indicate that Count, DDS, BI, EI and DQD were positively associated with daily energy intake with coefficients of 21·1, 133·2, 619·4, 231·7 and 89·3 (all *P* < 0·01), respectively. CHDI and CFPS were negatively correlated with daily energy intake with coefficients of −14·2 and −17·7, respectively (referring to Tables A5 and A6 in appendix A for detailed results).

**Table 9:** The associations between diet quality indices and daily energy intakes from ordinary least squares using individual/household cluster effect (*n* 30 350)

	Individual cluster effect*			Household cluster effect†		
	Coefficients	95 % CI	*P*	Coefficients	95 % CI	*P*
Count	21·1	19·5, 22·7	<0·01	21·1	18·9, 23·3	<0·01
DDS	133·4	124·6, 142·1	<0·01	133·4	122·1, 144·6	<0·01
BI	617·4	500·2, 734·6	<0·01	617·4	461·1, 773·7	<0·01
EI	231·6	209·6, 253·6	<0·01	231·6	202·4, 260·7	<0·01
CHDI	−14·2	−15·8, −12·7	<0·01	−14·2	−16·2, −12·3	<0·01
CFPS	−17·6	−25·4, −9·8	<0·01	−17·6	−27·5, −7·8	<0·01
DQD	89·2	84·9, 93·5	<0·01	89·2	84·1, 94·3	<0·01

*,†Denote the full estimated results could be found in Tables A5 and A6 in appendix A, respectively.

Associations are investigated by ordinary least squares linear regressions using individual/household cluster effects, taking account of gender, age, education, household net income per capita in logarithm, daily sedentary activity time, daily exercise time, labour intensity level, frequency of drinking alcohol, household size, urbanisation index and dummy variables for different years.

## Discussion

The results showed that the scores of positive measurements, including Count, DDS, BI, EI, CFPS and CHDI significantly increased (all *P* < 0·01) over 2004–2011, while the value of negative measurement (i.e. DQD) declined during the same period that manifests the improvement of diet quality for adults in China. Those results are consistent with the previous literature^(17,19)^. However, with the rapid development of social-economy, the fast income growth would follow a significant upgrading food consumption for Chinese^(1)^. The Chinese diets have been gradually shifting from traditional patterns dominated by cereals and vegetables to patterns with high proportions of meat, poultry, fat and small shares of staple foods^(1,2)^. Such transformation would not only increase the diversity of diets but also increase the intakes of energy and fat^(19,44)^. Moreover, the development of the food industry (e.g. fast-food industry) leads increasing meat, sugar, fat and energy density foods in Chinese diets, which may increase the risks for OW, DM and CVD^(2,17)^. According to the results in this study and previous literature, Chinese diets are predicted to be more diverse in the future^(1,2)^. However, diet-related NCD and risk for OW have become more prevalent in China^(45)^. Therefore, it is necessary for Chinese residents to be vigilant against high-energy-density foods (e.g. high-fat and high-sugar foods) and over-nutrition as diets becoming more diverse.

The results from mean-comparison tests revealed that participants with DM, MI and OW hold higher dietary diversity (namely, higher values of Count, DDS, BI and EI) than their counterparts, respectively. Furthermore, the binary Logit regressions also indicated that the count indices (i.e. Count and DDS) were significantly positively associated with the risk for OW. And the distribution indices (i.e. BI and EI) were also positively associated with DM, MI and the risk for OW. That is inconsistent with the general health expectation of diet quality. For example, the previous literature pointed out that high-quality diets are associated with optimal BMI^(18)^. One possible reason is that dietary diversity indices mainly take the number of food items and the average of food distribution into consideration but commonly override the information about food attributes and nutrition guidelines^(20)^. And higher dietary diversity might end up with excess food and energy intakes (e.g. consuming too much meat and fat) and over-nutrition as mentioned in some studies^(19)^. Therefore, there are caveats which concern the diet quality assessments with dietary diversity indices^(19,20,46,47)^. Furthermore, the empirical results from OLS models also showed that the dietary diversity indices were significantly positively associated with daily energy intake, which implies that higher dietary diversity may be accompanied by potential risks of over-nutrition^(19,47)^.

Comparing with dietary diversity indices, dietary guideline-based indices might be more appropriate indicators for measuring diet quality. The results from OLS models indicate that higher CHDI and CFPS, namely better diet quality, were significantly negatively correlated with daily energy intakes. And a larger DQD, indicating a worse diet quality, was significantly positively associated with daily energy intake. This implies diets that follow the dietary guidelines would avoid the potential risk of over-nutrition^(30,31)^. Furthermore, the results from Logit regressions indicated that the higher CHDI and CFPS scores were negatively associated with DM and risk for OW. It is consistent with the general expectation of that better diet quality would be followed by better health^(2,3,48)^. However, these two measurements were insignificantly correlated with MI. When it comes to DQD, the larger DQD, namely a worse diet quality, contributed to higher risks of OW, DM and MI that kept in line with the traditional expectation of nutrition transition^(2,31)^. All CHDI, CFPS and DQD measurements were composed based on CDG and took account of information on food variety, distribution and balanced diet in various degrees^(1,17,24)^. One reason for the differences of associations between various guideline-based indices and health risk factors might be the subjective nature of the cut-off weights applied in CHDI and CFPS^(17)^. Specifically, one concern associated with CHDI and CFPS is that those two measurements set different cut-off weights for various food groups in the process of index composing^(1,24,41)^. These cut-off weights may be influenced by subjective choices^(1,24)^, which would undermine the precision of the diet quality evaluation^(17)^. Meanwhile, DQD used the cumulative absolute divergence between dietary consumptions and recommendations from CFP 2016 to measure diet quality to avoid subjective cut-off weights setting for various food groups^(17)^. Therefore, DQD could be the relatively objective and comprehensive indicator among these seven diet quality measures in the present study, as it properly revealed the diet quality and its association with the NCD and risk for OW and daily energy intakes.

There is an interesting result in this study one might get confused. From 2004 to 2011, all diet quality indexes manifested the improvement of diet quality for Chinese adults, though OW, DM and MI became more prevalent. While according to Logit regressions results, better diet quality (i.e. CHDI, CFPS and DQD) was negatively associated with DM and risk for OW. One possible reason is that NCD and risk for OW are generally affected by many factors (e.g. diet quality, physical exercise and mood). Although both the average diet quality and the prevalence of OW and DM for Chinese adults showed an upward trend from 2004 to 2011, it should not necessarily mean that better CHDI, CFPS and DQD would associate with a higher prevalence of OW and NCD. To explore the associations between diet quality and NCD and risk for OW, Logit regressions with control for some other major factors affecting health status were performed. The regression results indicated that better CHDI, CFPS and DQD were associated with lower risks of OW and DM.

The associations between dietary guideline-based indices and NCD as well as risk for OW were generally keeping in line with the evidence in the previous literature and traditional health expectation that high-quality diets would end up with better health^(48,49)^. Therefore, those guideline-based indices should be ranked in higher levels than the diversity indices. Furthermore, DQD is generally more robust than the other six diet quality indices to some extent. All in all, taking account of the definitions, main features and healthy implications of the diet quality measurements based on empirical results, the seven indices in this study could be ranked in three levels: Rank I (Count, DDS, BI, EI), Rank II (CHDI, CFPS) and Rank III (DQD), and a higher rank implies a better performance of diet quality index.

It should bear in mind that even though DQD is a relatively robust indicator among these seven indices, it still has some imperfections. For example, DQD evaluates diet quality through the absolute value of divergence between dietary intakes and CFP 2016. This means it is essential to check the actual food intakes to determine whether the divergence is caused by over-nutrition or under-nutrition in some cases^(17)^. In addition, DQD does not distinguish the diet standards for male and female, and the estimations might be biased. Due to the complexity of food consumption and the imperfections of the available diet quality indices^(17,20)^, we need to look for more objective and comprehensive diet quality measurements in the future.

There are several limitations in the present study. Firstly, oil and salt were not included due to the data unavailability^(17)^. In addition, some processed foods (e.g. pre-prepared pizza and hamburger) are not included in the Chinese Food Composition Table (CFCT 2002–2004) as the specific ingredients are not clear^(36)^. Besides, following the original construction methods of the seven diet quality indices, the present study did not refer to the NOVA food classification system to distinguish the effects of unprocessed food and processed food on diet-related health. Based on the NOVA food classification system^(50)^, food items used to calculate diet quality in the present study belong to food group1 (unprocessed or minimally processed foods) or group3 (processed foods, like dried meat). Due to data unavailability, food group2 (i.e. oils, fats, salts, sugar) was excluded. In addition, formulations/ingredient of food in the group4 (ultra-processed food, like packaged snacks, biscuits, beverages) were unavailable in CHNS dataset. Intake of these ultra-processed foods could not be divided into intakes of specific food group (e.g. grains, eggs) to calculate diet quality. Therefore, food group4 was also not evolved to calculate diet quality.

Secondly, even though CHNS had put great efforts on data quality, dietary consumption data obtained through a 24-h recall method may still suffer measurement bias. Thirdly, due to limitation of space, only a few representative diet quality measurements were selected. Some other widely used indices were not included. For example, Chinese healthy eating index^(25)^ is a guideline-based indices similar to CHDI but not used in the present study. The systematic comparisons between more indicators are needed in near future. Besides, we only analysed DM, MI and OW, and it may not represent the whole health status. More types of indicators should be taken into consideration in further analysis. Fourthly, instead of cardiometabolic risk markers, reported NCD were used due to data unavailability that might underestimate the populations with NCD and then underestimate the impacts of diet quality on NCD. Besides, the present study could not distinguish type 1 and type 2 diabetes mellitus. Fifthly, potential endogeneity problem of the explanatory variables was not tackled, and the estimation might be biased. NCD and risk of OW can be affected by observed variables (e.g. income) and unobserved variables (e.g. personal character, family relationships, personal pressure). These variables might correlate with diet quality. Moreover, there might be reverse causality between these variables and nutrition-related health^(4)^. That means the estimated results in the present study can only be interpreted as correlations. To obtain precise results, more data and improved econometric modelling approaches should be employed in the next step. Sixthly, following the original construction method of CHDI, CFPS and DQD, the present study did not control the gender difference in calculating diet quality. Seventhly, given the complexity of relationships between diet consumption and health, this study only selected some major factors due to the availability of the data and may get some missing variables.

## Conclusions

This study employed four dietary diversity measurements (i.e. Count, DDS, BI and EI) and three dietary guideline-based measurements (i.e. CHDI, CFPS and DQD) from the previous literature to evaluate the dynamics of diet quality for Chinese adults and their different associations with NCD and risk for OW with the data from CHNS between 2004 and 2011. The results indicated that diet quality for Chinese adults generally improved over the period of 2004–2011. Chinese diets are expected to be more diverse in the future with the increasing income and diversifying food supply. Moreover, taking all the information and performance of the diet quality indices into consideration, seven selected indices in this study could be ranked in three levels: Rank I (Count, DDS, BI, EI), Rank II (CHDI, CFPS) and Rank III (DQD), and a higher rank implies a better performance of the index. The results also indicate that dietary guideline-based indices are more robust than dietary diversity indices in measuring diet quality. Dietary guideline-based indices could properly reveal the diet quality and its association with the NCD and risk for OW, while higher dietary diversity may usually be associated with over-nutrition. This implies that increasing diversity of the diet, although often advised, does not necessarily improve the nutrition and health. Dietary diversity is a necessary but not sufficient condition for a balanced diet. Researchers and policy makers should follow dietary guidelines as closely as possible in the process of diet quality evaluation. In addition, to improve Chinese diet quality and health in the future, we suggest that national healthy diet policies should pay more attention to health education and encourage residents to avoid over-nutrition when their diets are becoming more diverse.

## Figures and Tables

**Fig. A1.1:**
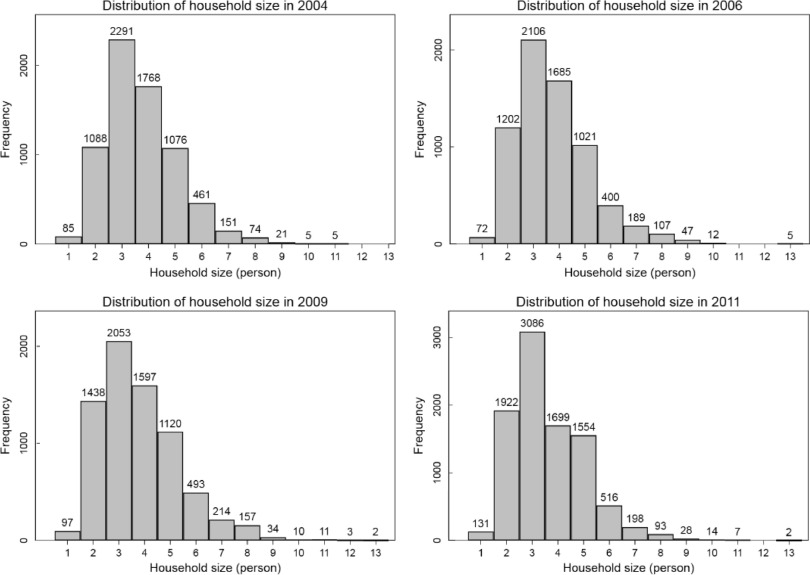
Distribution of household size across years

## Appendix A

**Table A1:** Average marginal effects of count and DDS on NCD and risk for OW from Logit regressions (*n* 30 350)

	OW		DM		MI		OW		DM		MI	
Cluster effect	Ind	Hh	Ind	Hh	Ind	Hh	Ind	Hh	Ind	Hh	Ind	Hh
Count	2·7 ^b^	2·7 ^b^	−3·5 ^d^	−3·5 ^d^	−1·8 ^d^	−1·8 ^d^						
P	<0·01	<0·01	0·83	0·83	0·83	0·83						
DDS							2·0 ^b^	2·0 ^b^	−1·4 ^b^	−1·4 ^b^	−1·4 ^c^	−1·4 ^c^
P							0·59	0·61	0·14	0·15	0·75	0·76
Gender ^1^	3·0 ^a^	3·0 ^a^	9·6 ^b^	9·6 ^b^	1·5 ^b^	1·5 ^b^	2·9 ^a^	2·9 ^a^	9·5 ^b^	9·5 ^b^	1·5 ^b^	1·5 ^b^
P	<0·01	<0·01	<0·01	<0·01	0·15	0·15	<0·01	<0·01	<0·01	<0·01	0·15	0·15
Age	5·9 ^b^	5·9 ^b^	1·8 ^b^	1·8 ^b^	5·0 ^c^	5·0 ^c^	5·9 ^b^	5·9 ^b^	1·8 ^b^	1·8 ^b^	5·0 ^c^	5·0 ^c^
P	<0·01	<0·01	<0·01	<0·01	<0·01	<0·01	<0·01	<0·01	<0·01	<0·01	<0·01	<0·01
Education ^2^												
Primary school	−1·0 ^a^	−1·0 ^a^	1·6 ^b^	1·6 ^b^	6·1 ^c^	6·1 ^c^	−9·7 ^b^	−9·7 ^b^	1·7 ^b^	1·7 ^b^	6·2 ^c^	6·2 ^c^
P	0·42	0·42	0·60	0·61	0·55	0·55	0·44	0·44	0·57	0·58	0·55	0·55
Lower middle school	−6·2 ^c^	−6·2 ^c^	5·0 ^b^	5·0 ^b^	1·3 ^b^	1·3 ^b^	6·7 ^c^	6·7 ^c^	5·2 ^b^	5·2 ^b^	1·3 ^b^	1·3 ^b^
P	0·96	0·96	0·08	0·08	0·23	0·23	0·96	0·96	0·06	0·07	0·23	0·23
Upper middle school	−2·2 ^a^	−2·2 ^a^	4·4 ^b^	4·4 ^b^	3·2 ^b^	3·2 ^b^	−2·0 ^a^	−2·0 ^a^	4·7 ^b^	4·7 ^b^	3·2 ^b^	3·2 ^b^
P	0·15	0·16	0·20	0·21	0·06	0·06	0·19	0·20	0·17	0·18	0·06	0·06
Vocational degree	−5·9 ^a^	−5·9 ^a^	−1·1 ^b^	−1·1 ^b^	−1·3 ^c^	−1·3 ^c^	−5·6 ^a^	−5·6 ^a^	−7·1 ^c^	−7·1 ^c^	−1·3 ^c^	−1·3 ^c^
P	<0·01	<0·01	0·77	0·77	0·93	0·93	<0·01	<0·01	0·85	0·85	0·94	0·94
University degree or higher	−6·8 ^a^	−6·8 ^a^	5·3 ^b^	5·3 ^b^	3·8 ^b^	3·8 ^b^	−6·4 ^a^	−6·4 ^a^	5·8 ^b^	5·8 ^b^	3·8 ^b^	3·8 ^b^
P	<0·01	<0·01	0·22	0·23	0·14	0·15	<0·01	<0·01	0·19	0·19	0·15	0·15
Income ^3^	8·0 ^b^	8·0 ^b^	1·6 ^b^	1·6 ^b^	−1·6 ^c^	−1·6 ^c^	1·0 ^a^	1·0 ^a^	1·8 ^b^	1·8 ^b^	−1·5 ^c^	−1·5 ^c^
P	0·02	0·03	0·12	0·11	0·74	0·75	<0·01	0·01	0·08	0·08	0·75	0·75
Sedentary ^4^	−1·1 ^b^	−1·1 ^b^	2·3 ^c^	2·3 ^c^	1·7 ^c^	1·7 ^c^	−7·7 ^c^	−7·7 ^c^	2·6 ^c^	2·6 ^c^	1·7 ^c^	1·7 ^c^
P	0·42	0·42	0·53	0·52	0·21	0·21	0·58	0·58	0·47	0·47	0·19	0·19
Exercise ^5^	5·4 ^b^	5·4 ^b^	−2·4 ^b^	−2·4 ^b^	4·9 ^c^	4·9 ^c^	6·6 ^b^	6·6 ^b^	−2·3 ^b^	−2·3 ^b^	5·0 ^c^	5·0 ^c^
P	0·38	0·38	0·10	0·10	0·28	0·32	0·29	0·29	0·12	0·12	0·27	0·30
Labour intensity ^6^												
Moderate	−7·4 ^b^	−7·4 ^b^	−5·9 ^b^	−5·9 ^b^	−2·7 ^b^	−2·7 ^b^	−8·4 ^b^	−8·4 ^b^	−6·1 ^b^	−6·1 ^b^	−2·7 ^b^	−2·7 ^b^
P	0·43	0·43	0·04	0·05	0·02	0·02	0·36	0·37	0·04	0·04	0·02	0·02
Heavy	−6·4 ^a^	−6·4 ^a^	−1·2 ^a^	−1·2 ^a^	−1·5 ^b^	−1·5 ^b^	−6·7 ^a^	−6·7 ^a^	−1·2 ^a^	−1·2 ^a^	−1·5 ^b^	−1·5 ^b^
P	<0·01	<0·01	<0·01	<0·01	0·22	0·22	<0·01	<0·01	<0·01	<0·01	0·22	0·22
Drinking ^7^												
Very low	−5·3 ^b^	−5·3 ^b^	−5·9 ^b^	−5·9 ^b^	−2·2 ^b^	−2·2 ^b^	−3·3 ^b^	−3·3 ^b^	−5·8 ^b^	−5·8 ^b^	−2·2 ^b^	−2·2 ^b^
P	0·72	0·71	0·09	0·09	0·13	0·13	0·82	0·82	0·10	0·09	0·13	0·13
Low	3·5 ^a^	3·5 ^a^	−6·6 ^b^	−6·6 ^b^	3·8 ^c^	3·8 ^c^	3·7 ^a^	3·7 ^a^	−6·4 ^b^	−6·4 ^b^	3·8 ^c^	3·8 ^c^
P	<0·01	<0·01	0·04	0·04	0·83	0·83	<0·01	<0·01	0·04	0·04	0·83	0·83
Medium	7·5 ^a^	7·5 ^a^	−1·3 ^b^	−1·3 ^b^	−2·3 ^b^	−2·3 ^b^	7·8 ^a^	7·8 ^a^	−1·1 ^b^	−1·1 ^b^	−2·3 ^b^	−2·3 ^b^
P	<0·01	<0·01	0·71	0·71	0·06	0·06	<0·01	<0·01	0·74	0·75	0·06	0·06
High	8·5 ^a^	8·5 ^a^	−9·0 ^b^	−9·0 ^b^	−3·8 ^b^	−3·8 ^b^	8·7 ^a^	8·7 ^a^	−9·0 ^b^	−9·0 ^b^	−3·9 ^b^	−3·9 ^b^
P	<0·01	<0·01	0·01	0·01	<0·01	<0·01	<0·01	<0·01	0·01	0·01	<0·01	<0·01
Very high	4·9 ^b^	4·9 ^b^	−1·1 ^a^	−1·1 ^a^	−2·4 ^b^	−2·4 ^b^	8·2 ^b^	8·2 ^b^	−1·1 ^a^	−1·1 ^a^	−2·4 ^b^	−2·4 ^b^
P	0·72	0·72	<0·01	<0·01	0·03	0·03	0·54	0·54	<0·01	<0·01	0·02	0·02
Smoking	−3·0 ^b^	−3·0 ^b^	−3·2 ^c^	−3·2 ^c^	−9·3 ^d^	−9·3 ^d^	−3·1 ^b^	−3·1 ^b^	−3·2 ^c^	−3·2 ^c^	−9·3 ^d^	−9·3 ^d^
P	<0·01	<0·01	0·02	0·02	0·18	0·18	<0·01	<0·01	0·02	0·02	0·18	0·18
Household size	−1·3 ^a^	−1·3 ^a^	−1·5 ^b^	−1·5 ^b^	−3·2 ^c^	−3·2 ^c^	−1·3 ^a^	−1·3 ^a^	−1·5 ^b^	−1·5 ^b^	−3·3 ^c^	−3·3 ^c^
P	<0·01	<0·01	0·03	0·03	0·27	0·27	<0·01	<0·01	0·02	0·02	0·26	0·25
Urbanisation ^8^	3·3 ^c^	3·3 ^c^	3·0 ^c^	3·0 ^c^	−1·1 ^d^	−1·1 ^d^	5·5 ^c^	5·5 ^c^	3·1 ^c^	3·1 ^c^	−1·0 ^d^	−1·0 ^d^
P	0·21	0·24	<0·01	<0·01	0·73	0·73	0·03	0·04	<0·01	<0·01	0·73	0·73
2006	5·9 ^b^	5·9 ^b^	3·8 ^b^	3·8 ^b^	8·6 ^c^	8·6 ^c^	7·1 ^b^	7·1 ^b^	3·9 ^b^	3·9 ^b^	8·7 ^c^	8·7 ^c^
P	0·32	0·33	0·14	0·14	0·47	0·47	0·23	0·25	0·13	0·13	0·47	0·47
2009	2·4 ^a^	2·4 ^a^	1·1 ^a^	1·1 ^a^	2·7 ^b^	2·7 ^b^	2·6 ^a^	2·6 ^a^	1·1 ^a^	1·1 ^a^	2·7 ^b^	2·7 ^b^
P	<0·01	<0·01	<0·01	<0·01	0·02	0·02	<0·01	<0·01	<0·01	<0·01	0·02	0·02
2011	4·7 ^a^	4·7 ^a^	1·3 ^a^	1·3 ^a^	5·7 ^d^	5·7 ^d^	5·4 ^a^	5·4 ^a^	1·3 ^a^	1·3 ^a^	3·3 ^d^	3·3 ^d^
P	<0·01	<0·01	<0·01	<0·01	0·96	0·96	<0·01	<0·01	<0·01	<0·01	0·98	0·98
Wald *χ* ^2^(23)	900·29	849·84	704·45	695·71	218·41	221·72	886·57	840·26	703·54	695·13	218·5	223·19
*P* value > *χ* ^2^	<0·01	<0·01	<0·01	<0·01	<0·01	<0·01	<0·01	<0·01	<0·01	<0·01	<0·01	<0·01
Pseudo *R*-square	0·03	0·03	0·15	0·15	0·13	0·13	0·03	0·03	0·15	0·15	0·13	0·13
Correctly classified (%)	61·1	61·1	98·1	98·1	99·6	99·6	61·1	61·1	98·1	98·1	99·6	99·6

OW, DM and MI are acronyms of overweight, diabetes mellitus and myocardial infarction, respectively. Overweight is defined as BMI ≥ 24 kg/m^2^. Ind denotes individual cluster effect; Hh denotes household cluster effect. ^1^ Gender: 1 = male, 0 = female; ^2^ Highest education completed: 1 = no school completed (reference group), 2 = primary school, 3 = lower middle school, 4 = upper middle school, 5 = vocational degree, 6 = undergraduate or higher degrees; ^3^ The annual household net income per capita after deflation at 2015 prices and then taking natural logarithm; ^4^ Daily sedentary activity time (hours); ^5^ Daily physical exercise time (minutes); ^6^ Labour intensity levels: 1 = light physical activity (reference category), working in a sitting or standing position (e.g. office work, counter salesperson); 2 = moderate physical activity (e.g. driver, electrician); 3 = heavy physical activity (e.g. farmer, athlete, steel worker, lumber worker); ^7^ The frequency of drinking alcohol, 1 = no drinking (reference group); 2 = very low frequency, no more than once a month; 3 = low frequency, once or twice a month; 4 = medium frequency, once or twice a week; 5 = high frequency, 3–4 times a week; 6 = very high frequency, almost every day; ^8^ Defined by a multidimensional 12-component urbanisation index, including the population density, physical, social, cultural and economic environment. ^a^ The coefficient is displayed in scientific notation format: coefficient × 10^−2^; ^b^ scientific notation format: coefficient × 10^−3^; ^c^ scientific notation format: coefficient × 10^−4^; ^d^ scientific notation format: coefficient × 10^−5^.

**Table A2:** Average marginal effects of BI and EI on NCD and risk for OW from Logit regressions (*n* 30 350)

	OW		DM		MI		OW		DM		MI	
Cluster effect	Ind	Hh	Ind	Hh	Ind	Hh	Ind	Hh	Ind	Hh	Ind	Hh
BI	0·3	0·3	8·1 ^b^	8·1 ^b^	1·2 ^a^	1·2 ^a^						
P	<0·01	<0·01	0·64	0·64	0·08	0·08						
EI							5·7 ^a^	5·7 ^a^	1·8 ^b^	1·8 ^b^	1·1 ^b^	1·1 ^b^
P							<0·01	<0·01	0·51	0·51	0·31	0·33
Gender ^1^	3·2 ^a^	3·2 ^a^	9·7 ^b^	9·7 ^b^	1·6 ^b^	1·6 ^b^	3·2 ^a^	3·2 ^a^	9·7 ^b^	9·7 ^b^	1·6 ^b^	1·6 ^b^
P	<0·01	<0·01	<0·01	<0·01	0·13	0·13	<0·01	<0·01	<0·01	<0·01	0·14	0·14
Age	5·9 ^b^	5·9 ^b^	1·8 ^b^	1·8 ^b^	5·0 ^c^	5·0 ^c^	5·9 ^b^	5·9 ^b^	1·8 ^b^	1·8 ^b^	5·0 ^c^	5·0 ^c^
P	<0·01	<0·01	<0·01	<0·01	<0·01	<0·01	<0·01	<0·01	<0·01	<0·01	<0·01	<0·01
Education ^2^												
Primary school	−1·1 ^a^	−1·1 ^a^	1·5 ^b^	1·5 ^b^	5·4 ^c^	5·4 ^c^	−1·1 ^a^	−1·1 ^a^	1·5 ^b^	1·5 ^b^	5·7 ^c^	5·7 ^c^
P	0·38	0·38	0·62	0·63	0·60	0·60	0·37	0·37	0·63	0·63	0·58	0·58
Lower middle school	−1·8 ^b^	−1·8 ^b^	4·8 ^b^	4·8 ^b^	1·1 ^b^	1·1 ^b^	−2·4 ^b^	−2·4 ^b^	4·8 ^b^	4·8 ^b^	1·2 ^b^	1·2 ^b^
P	0·89	0·89	0·09	0·10	0·29	0·29	0·85	0·86	0·10	0·10	0·27	0·27
Upper middle school	−2·3 ^a^	−2·3 ^a^	4·2 ^b^	4·2 ^b^	3·0 ^b^	3·0 ^b^	−2·4 ^a^	−2·4 ^a^	4·1 ^b^	4·1 ^b^	3·1 ^b^	3·1 ^b^
P	0·13	0·14	0·22	0·23	0·08	0·08	0·12	0·12	0·23	0·24	0·07	0·07
Vocational degree	−5·9 ^a^	−5·9 ^a^	−1·3 ^b^	−1·3 ^b^	−2·6 ^c^	−2·6 ^c^	−6·0 ^a^	−6·0 ^a^	−1·4 ^b^	−1·4 ^b^	−2·4 ^c^	−2·4 ^c^
P	<0·01	<0·01	0·73	0·74	0·87	0·87	<0·01	<0·01	0·72	0·72	0·88	0·88
University degree or higher	−6·6 ^a^	−6·6 ^a^	5·1 ^b^	5·1 ^b^	3·6 ^b^	3·6 ^b^	−6·9 ^a^	−6·9 ^a^	5·0 ^b^	5·0 ^b^	3·6 ^b^	3·6 ^b^
P	<0·01	<0·01	0·24	0·24	0·16	0·16	<0·01	<0·01	0·25	0·25	0·16	0·16
Income ^3^	7·7 ^b^	7·7 ^b^	1·5 ^b^	1·5 ^b^	−2·6 ^c^	−2·6 ^c^	6·9 ^b^	6·9 ^b^	1·4 ^b^	1·4 ^b^	−2·4 ^c^	−2·4 ^c^
P	0·03	0·04	0·14	0·14	0·55	0·56	0·05	0·06	0·15	0·15	0·60	0·61
Sedentary ^4^	−1·1 ^b^	−1·1 ^b^	2·1 ^c^	2·1 ^c^	1·5 ^c^	1·5 ^c^	−1·2 ^b^	−1·2 ^b^	2·1 ^c^	2·1 ^c^	1·6 ^c^	1·6 ^c^
P	0·42	0·43	0·56	0·55	0·25	0·26	0·39	0·39	0·57	0·56	0·24	0·25
Exercise ^5^	6·0 ^b^	6·0 ^b^	−2·5 ^b^	−2·5 ^b^	4·5 ^c^	4·5 ^c^	5·5 ^b^	5·5 ^b^	−2·5 ^b^	−2·5 ^b^	4·5 ^c^	4·5 ^c^
P	0·33	0·33	0·10	0·10	0·33	0·36	0·37	0·38	0·09	0·09	0·33	0·36
Labour intensity ^6^												
Moderate	−7·1 ^b^	−7·1 ^b^	−5·8 ^b^	−5·8 ^b^	−2·6 ^b^	−2·6 ^b^	−6·5 ^b^	−6·5 ^b^	−5·8 ^b^	−5·8 ^b^	−2·6 ^b^	−2·6 ^b^
P	0·44	0·45	0·05	0·05	0·02	0·02	0·48	0·49	0·05	0·05	0·02	0·02
Heavy	−6·2 ^a^	−6·2 ^a^	−1·2 ^a^	−1·2 ^a^	−1·3 ^b^	−1·3 ^b^	−6·1 ^a^	−6·1 ^a^	−1·2 ^a^	−1·2 ^a^	−1·4 ^b^	−1·4 ^b^
P	<0·01	<0·01	<0·01	<0·01	0·29	0·29	<0·01	<0·01	<0·01	<0·01	0·26	0·26
Drinking ^7^												
Very low	−3·1 ^b^	−3·1 ^b^	−5·9 ^b^	−5·9 ^b^	−2·3 ^b^	−2·3 ^b^	−4·5 ^b^	−4·5 ^b^	−5·9 ^b^	−5·9 ^b^	−2·3 ^b^	−2·3 ^b^
P	0·83	0·83	0·09	0·08	0·12	0·12	0·76	0·76	0·09	0·08	0·12	0·12
Low	3·6 ^a^	3·6 ^a^	−6·6 ^b^	−6·6 ^b^	3·2 ^c^	3·2 ^c^	3·5 ^a^	3·5 ^a^	−6·7 ^b^	−6·7 ^b^	3·2 ^c^	3·2 ^c^
P	<0·01	<0·01	0·03	0·03	0·85	0·85	<0·01	<0·01	0·03	0·03	0·85	0·85
Medium	7·6 ^a^	7·6 ^a^	−1·4 ^b^	−1·4 ^b^	−2·4 ^b^	−2·4 ^b^	7·5 ^a^	7·5 ^a^	−1·4 ^b^	−1·4 ^b^	−2·4 ^b^	−2·4 ^b^
P	<0·01	<0·01	0·69	0·69	0·05	0·05	<0·01	<0·01	0·68	0·68	0·06	0·06
High	8·5 ^a^	8·5 ^a^	−9·1 ^b^	−9·1 ^b^	−3·9 ^b^	−3·9 ^b^	8·4 ^a^	8·4 ^a^	−9·2 ^b^	−9·2 ^b^	−3·9 ^b^	−3·9 ^b^
P	<0·01	<0·01	0·01	0·01	<0·01	<0·01	<0·01	<0·01	0·01	0·01	<0·01	<0·01
Very high	5·4 ^b^	5·4 ^b^	−1·1 ^a^	−1·1 ^a^	−2·4 ^b^	−2·4 ^b^	4·1 ^b^	4·1 ^b^	−1·1 ^a^	−1·1 ^a^	−2·4 ^b^	−2·4 ^b^
P	0·69	0·69	<0·01	<0·01	0·02	0·02	0·76	0·76	<0·01	<0·01	0·02	0·02
Smoking	−3·1 ^b^	−3·1 ^b^	−3·2 ^c^	−3·2 ^c^	−9·3 ^d^	−9·3 ^d^	−3·1 ^b^	−3·1 ^b^	−3·2 ^c^	−3·2 ^c^	−9·3 ^d^	−9·3 ^d^
P	<0·01	<0·01	0·02	0·02	0·17	0·17	<0·01	<0·01	0·02	0·02	0·18	0·18
Household size	−1·2 ^a^	−1·2 ^a^	−1·5 ^b^	−1·5 ^b^	−3·1 ^c^	−3·1 ^c^	−1·3 ^a^	−1·3 ^a^	−1·5 ^b^	−1·5 ^b^	−3·4 ^c^	−3·4 ^c^
P	<0·01	<0·01	0·03	0·03	0·28	0·28	<0·01	<0·01	0·02	0·02	0·24	0·24
Urbanisation ^8^	2·6 ^c^	2·6 ^c^	2·9 ^c^	2·9 ^c^	−2·6 ^d^	−2·6 ^d^	1·7 ^c^	1·7 ^c^	2·8 ^c^	2·8 ^c^	−2·1 ^d^	−2·1 ^d^
P	0·32	0·34	<0·01	<0·01	0·40	0·41	0·52	0·55	<0·01	<0·01	0·51	0·52
2006	4·1 ^b^	4·1 ^b^	3·8 ^b^	3·8 ^b^	7·4 ^c^	7·4 ^c^	3·9 ^b^	3·9 ^b^	3·8 ^b^	3·8 ^b^	8·0 ^c^	8·0 ^c^
P	0·49	0·50	0·15	0·15	0·54	0·54	0·52	0·53	0·15	0·15	0·51	0·51
2009	2·1 ^a^	2·1 ^a^	1·1 ^a^	1·1 ^a^	2·5 ^b^	2·5 ^b^	2·0 ^a^	2·0 ^a^	1·1 ^a^	1·1 ^a^	2·6 ^b^	2·6 ^b^
P	<0·01	<0·01	<0·01	<0·01	0·03	0·03	<0·01	0·01	<0·01	<0·01	0·03	0·02
2011	4·7 ^a^	4·7 ^a^	1·3 ^a^	1·3 ^a^	−2·9 ^c^	−2·9 ^c^	4·4 ^a^	4·4 ^a^	1·3 ^a^	1·3 ^a^	−2·0 ^c^	−2·0 ^c^
P	<0·01	<0·01	<0·01	<0·01	0·80	0·80	<0·01	<0·01	<0·01	<0·01	0·86	0·86
Wald *χ* ^2^(23)	914·53	855·07	704·6	695·74	212·67	214·9	918·02	860·7	704·88	695·83	218·89	222·5
P value > *χ* ^2^	<0·01	<0·01	<0·01	<0·01	<0·01	<0·01	<0·01	<0·01	<0·01	<0·01	<0·01	<0·01
Pseudo *R*-square	0·03	0·03	0·15	0·15	0·13	0·13	0·03	0·03	0·15	0·15	0·13	0·13
Correctly classified (%)	61·1	61·1	98·1	98·1	99·6	99·6	61·2	61·2	98·1	98·1	99·6	99·6

OW, DM and MI are acronyms of overweight, diabetes mellitus and myocardial infarction, respectively. Overweight is defined as BMI ≥ 24 kg/m^2^. Ind denotes individual cluster effect; Hh denotes household cluster effect. ^1^ Gender: 1 = male, 0 = female; ^2^ Highest education completed: 1 = no school completed (reference group), 2 = primary school, 3 = lower middle school, 4 = upper middle school, 5 = vocational degree, 6 = undergraduate or higher degrees; ^3^ The annual household net income per capita after deflation at 2015 prices and then taking natural logarithm; ^4^ Daily sedentary activity time (hours); ^5^ Daily physical exercise time (minutes); ^6^ Labour intensity levels: 1 = light physical activity (reference category), working in a sitting or standing position (e.g. office work, counter salesperson); 2 = moderate physical activity (e.g. driver, electrician); 3 = heavy physical activity (e.g. farmer, athlete, steel worker, lumber worker); ^7^ The frequency of drinking alcohol, 1 = no drinking (reference group); 2 = very low frequency, no more than once a month; 3 = low frequency, once or twice a month; 4 = medium frequency, once or twice a week; 5 = high frequency, 3–4 times a week; 6 = very high frequency, almost every day; ^8^ Defined by a multidimensional 12-component urbanisation index, including the population density, physical, social, cultural and economic environment. ^a^ The coefficient is displayed in scientific notation format: coefficient × 10^−2^; ^b^ scientific notation format: coefficient × 10^−3^; ^c^ scientific notation format: coefficient × 10^−4^; ^d^ scientific notation format: coefficient × 10^−5^.

**Table A3:** Average marginal effects of CHDI and CFPS on NCD and risk for OW from Logit regressions (*n* 30 350)

	OW		DM		MI		OW		DM		MI	
Cluster effect	Ind	Hh	Ind	Hh	Ind	Hh	Ind	Hh	Ind	Hh	Ind	Hh
CHDI	−1·2 ^b^	−1·2 ^b^	−5·1 ^d^	−5·1 ^d^	7·5 ^d^	7·5 ^d^						
P	0·04	0·05	0·73	0·72	0·27	0·27						
CFPS							−1·3 ^a^	−1·3 ^a^	−1·1 ^b^	−1·1 ^b^	9·7 ^d^	9·7 ^d^
P							<0·01	<0·01	0·18	0·18	0·81	0·82
Gender ^1^	2·9 ^a^	2·9 ^a^	9·6 ^b^	9·6 ^b^	1·6 ^b^	1·6 ^b^	2·7 ^a^	2·7 ^a^	9·4 ^b^	9·4 ^b^	1·6 ^b^	1·6 ^b^
P	<0·01	<0·01	<0·01	<0·01	0·14	0·14	0·01	0·01	<0·01	<0·01	0·15	0·14
Age	5·9 ^b^	5·9 ^b^	1·8 ^b^	1·8 ^b^	5·0 ^c^	5·0 ^c^	5·9 ^b^	5·9 ^b^	1·8 ^b^	1·8 ^b^	5·0 ^c^	5·0 ^c^
P	<0·01	<0·01	<0·01	<0·01	<0·01	<0·01	<0·01	<0·01	<0·01	<0·01	<0·01	<0·01
Education ^2^												
Primary school	−9·3 ^b^	−9·3 ^b^	1·5 ^b^	1·5 ^b^	6·0 ^c^	6·0 ^c^	−7·9 ^b^	−7·9 ^b^	1·7 ^b^	1·7 ^b^	6·0 ^c^	6·0 ^c^
P	0·46	0·46	0·61	0·61	0·56	0·56	0·53	0·53	0·58	0·59	0·56	0·56
Lower middle school	1·2 ^b^	1·2 ^b^	5·0 ^b^	5·0 ^b^	1·2 ^b^	1·2 ^b^	3·0 ^b^	3·0 ^b^	5·1 ^b^	5·1 ^b^	1·3 ^b^	1·3 ^b^
P	0·92	0·92	0·08	0·08	0·24	0·24	0·81	0·82	0·07	0·07	0·24	0·24
Upper middle school	−1·9 ^a^	−1·9 ^a^	4·3 ^b^	4·3 ^b^	3·2 ^b^	3·2 ^b^	−1·7 ^a^	−1·7 ^a^	4·6 ^b^	4·6 ^b^	3·2 ^b^	3·2 ^b^
P	0·20	0·21	0·21	0·21	0·07	0·07	0·26	0·27	0·18	0·19	0·07	0·07
Vocational degree	−5·5 ^a^	−5·5 ^a^	−1·1 ^b^	−1·1 ^b^	−1·7 ^c^	−1·7 ^c^	−5·3 ^a^	−5·3 ^a^	−9·9 ^c^	−9·9 ^c^	−1·6 ^c^	−1·6 ^c^
P	<0·01	<0·01	0·76	0·76	0·91	0·92	<0·01	<0·01	0·79	0·79	0·92	0·92
University degree or higher	−6·3 ^a^	−6·3 ^a^	5·2 ^b^	5·2 ^b^	3·8 ^b^	3·8 ^b^	−6·0 ^a^	−6·0 ^a^	5·5 ^b^	5·5 ^b^	3·7 ^b^	3·7 ^b^
P	<0·01	<0·01	0·23	0·23	0·15	0·15	<0·01	<0·01	0·20	0·21	0·15	0·16
Income ^3^	1·1 ^a^	1·1 ^a^	1·6 ^b^	1·6 ^b^	−2·0 ^c^	−2·0 ^c^	1·2 ^a^	1·2 ^a^	1·7 ^b^	1·7 ^b^	−1·9 ^c^	−1·9 ^c^
P	<0·01	<0·01	0·12	0·12	0·66	0·66	<0·01	<0·01	0·10	0·10	0·68	0·69
Sedentary ^4^	−7·4 ^c^	−7·4 ^c^	2·2 ^c^	2·2 ^c^	1·7 ^c^	1·7 ^c^	−6·3 ^c^	−6·3 ^c^	2·4 ^c^	2·4 ^c^	1·7 ^c^	1·7 ^c^
P	0·59	0·60	0·54	0·53	0·20	0·21	0·65	0·65	0·52	0·51	0·21	0·21
Exercise ^5^	6·8 ^b^	6·8 ^b^	−2·4 ^b^	−2·4 ^b^	4·7 ^c^	4·7 ^c^	7·3 ^b^	7·3 ^b^	−2·4 ^b^	−2·4 ^b^	4·8 ^c^	4·8 ^c^
P	0·27	0·27	0·10	0·10	0·30	0·34	0·24	0·24	0·11	0·11	0·28	0·32
Labour intensity ^6^												
Moderate	−8·5 ^b^	−8·5 ^b^	−5·9 ^b^	−5·9 ^b^	−2·7 ^b^	−2·7 ^b^	−8·8 ^b^	−8·8 ^b^	−5·9 ^b^	−5·9 ^b^	−2·7 ^b^	−2·7 ^b^
P	0·36	0·36	0·05	0·05	0·02	0·02	0·34	0·35	0·05	0·05	0·02	0·02
Heavy	−6·8 ^a^	−6·8 ^a^	−1·2 ^a^	−1·2 ^a^	−1·5 ^b^	−1·5 ^b^	−6·9 ^a^	−6·9 ^a^	−1·2 ^a^	−1·2 ^a^	−1·5 ^b^	−1·5 ^b^
P	<0·01	<0·01	<0·01	<0·01	0·23	0·23	<0·01	<0·01	<0·01	<0·01	0·23	0·23
Drinking ^7^												
Very low	−3·1 ^b^	−3·1 ^b^	−5·9 ^b^	−5·9 ^b^	−2·3 ^b^	−2·3 ^b^	−2·9 ^b^	−2·9 ^b^	−5·8 ^b^	−5·8 ^b^	−2·3 ^b^	−2·3 ^b^
P	0·83	0·83	0·09	0·08	0·13	0·13	0·84	0·84	0·09	0·09	0·13	0·13
Low	3·7 ^a^	3·7 ^a^	−6·6 ^b^	−6·6 ^b^	3·7 ^c^	3·7 ^c^	3·7 ^a^	3·7 ^a^	−6·6 ^b^	−6·6 ^b^	3·6 ^c^	3·6 ^c^
P	<0·01	<0·01	0·04	0·03	0·83	0·83	<0·01	<0·01	0·04	0·03	0·83	0·83
Medium	7·8 ^a^	7·8 ^a^	−1·3 ^b^	−1·3 ^b^	−2·3 ^b^	−2·3 ^b^	7·8 ^a^	7·8 ^a^	−1·4 ^b^	−1·4 ^b^	−2·3 ^b^	−2·3 ^b^
P	<0·01	<0·01	0·70	0·70	0·06	0·06	<0·01	<0·01	0·70	0·70	0·06	0·06
High	8·8 ^a^	8·8 ^a^	−9·1 ^b^	−9·1 ^b^	−3·9 ^b^	−3·9 ^b^	8·8 ^a^	8·8 ^a^	−9·0 ^b^	−9·0 ^b^	−3·9 ^b^	−3·9 ^b^
P	<0·01	<0·01	0·01	0·01	<0·01	<0·01	<0·01	<0·01	0·01	0·01	<0·01	<0·01
Very high	8·5 ^b^	8·5 ^b^	−1·1 ^a^	−1·1 ^a^	−2·4 ^b^	−2·4 ^b^	9·1 ^b^	9·1 ^b^	−1·1 ^a^	−1·1 ^a^	−2·4 ^b^	−2·4 ^b^
P	0·53	0·53	<0·01	<0·01	0·02	0·02	0·50	0·50	<0·01	<0·01	0·02	0·02
Smoking	−3·1 ^b^	−3·1 ^b^	−3·2 ^c^	−3·2 ^c^	−9·3 ^d^	−9·3 ^d^	−3·1 ^b^	−3·1 ^b^	−3·2 ^c^	−3·2 ^c^	−9·3 ^d^	−9·3 ^d^
P	<0·01	<0·01	0·02	0·02	0·18	0·18	<0·01	<0·01	0·02	0·02	0·18	0·18
Household size	−1·2 ^a^	−1·2 ^a^	−1·5 ^b^	−1·5 ^b^	−3·4 ^c^	−3·4 ^c^	−1·3 ^a^	−1·3 ^a^	−1·5 ^b^	−1·5 ^b^	−3·3 ^c^	−3·3 ^c^
P	<0·01	<0·01	0·03	0·03	0·24	0·24	<0·01	<0·01	0·03	0·03	0·26	0·26
Urbanisation ^8^	6·2 ^c^	6·2 ^c^	3·0 ^c^	3·0 ^c^	−1·6 ^d^	−1·6 ^d^	7·1 ^c^	7·1 ^c^	3·1 ^c^	3·1 ^c^	−1·4 ^d^	−1·4 ^d^
P	0·01	0·02	<0·01	<0·01	0·59	0·59	0·01	0·01	<0·01	<0·01	0·64	0·64
2006	8·0 ^b^	8·0 ^b^	3·9 ^b^	3·9 ^b^	8·1 ^c^	8·1 ^c^	8·5 ^b^	8·5 ^b^	3·9 ^b^	3·9 ^b^	8·5 ^c^	8·5 ^c^
P	0·18	0·19	0·14	0·13	0·50	0·50	0·15	0·17	0·13	0·13	0·48	0·48
2009	2·7 ^a^	2·7 ^a^	1·1 ^a^	1·1 ^a^	2·6 ^b^	2·6 ^b^	2·8 ^a^	2·8 ^a^	1·1 ^a^	1·1 ^a^	2·7 ^b^	2·7 ^b^
P	<0·01	<0·01	<0·01	<0·01	0·02	0·02	<0·01	<0·01	<0·01	<0·01	0·02	0·02
2011	5·6 ^a^	5·6 ^a^	1·3 ^a^	1·3 ^a^	−7·6 ^d^	−7·6 ^d^	5·6 ^a^	5·6 ^a^	1·3 ^a^	1·3 ^a^	−5·0 ^e^	−5·0 ^e^
P	<0·01	<0·01	<0·01	<0·01	0·95	0·95	<0·01	<0·01	<0·01	<0·01	1·00	1·00
Wald *χ* ^2^(23)	890·92	843·69	704·04	695·66	210·61	210·7	902·18	850·5	707·23	698·5	208·39	209·03
P value > *χ* ^2^	<0·01	<0·01	<0·01	<0·01	<0·01	<0·01	<0·01	<0·01	<0·01	<0·01	<0·01	<0·01
Pseudo *R*-square	0·03	0·03	0·15	0·15	0·13	0·13	0·03	0·03	0·15	0·15	0·13	0·13
Correctly classified (%)	61·1	61·1	98·1	98·1	99·6	99·6	61·1	61·1	98·1	98·1	99·6	99·6

OW, DM and MI are acronyms of overweight, diabetes mellitus and myocardial infarction, respectively. Overweight is defined as BMI ≥ 24 kg/m^2^. Ind denotes individual cluster effect; Hh denotes household cluster effect. ^1^ Gender: 1 = male, 0 = female; ^2^ Highest education completed: 1 = no school completed (reference group), 2 = primary school, 3 = lower middle school, 4 = upper middle school, 5 = vocational degree, 6 = undergraduate or higher degrees; ^3^ The annual household net income per capita after deflation at 2015 prices and then taking natural logarithm; ^4^ Daily sedentary activity time (hours); ^5^ Daily physical exercise time (minutes); ^6^ Labour intensity levels: 1 = light physical activity (reference category), working in a sitting or standing position (e.g. office work, counter salesperson); 2 = moderate physical activity (e.g. driver, electrician); 3 = heavy physical activity (e.g. farmer, athlete, steel worker, lumber worker); ^7^ The frequency of drinking alcohol, 1 = no drinking (reference group); 2 = very low frequency, no more than once a month; 3 = low frequency, once or twice a month; 4 = medium frequency, once or twice a week; 5 = high frequency, 3–4 times a week; 6 = very high frequency, almost every day; ^8^ Defined by a multidimensional 12-component urbanisation index, including the population density, physical, social, cultural and economic environment. ^a^ The coefficient is displayed in scientific notation format: coefficient × 10^−2^; ^b^ scientific notation format: coefficient × 10^−3^; ^c^ scientific notation format: coefficient × 10^−4^; ^d^ scientific notation format: coefficient × 10^−5^; ^e^ scientific notation format: coefficient × 10^−6^.

**Table A4:** Average marginal effects of DQD on NCD and risk for OW from Logit regressions (*n* 30 350)

	OW		DM		MI	
Cluster effect	Ind	Hh	Ind	Hh	Ind	Hh
DQD	5·1 ^b^	5·1 ^b^	7·7 ^c^	7·7 ^c^	2·8 ^c^	2·8 ^c^
P	<0·01	<0·01	0·01	0·01	0·07	0·07
Gender ^1^	2·7 ^a^	2·7 ^a^	9·4 ^b^	9·4 ^b^	1·5 ^b^	1·5 ^b^
P	0·01	0·01	<0·01	<0·01	0·16	0·16
Age	5·9 ^b^	5·9 ^b^	1·8 ^b^	1·8 ^b^	5·0 ^c^	5·0 ^c^
P	<0·01	<0·01	<0·01	<0·01	<0·01	<0·01
Education ^2^						
Primary school	−8·8 ^b^	−8·8 ^b^	1·6 ^b^	1·6 ^b^	6·2 ^c^	6·2 ^c^
P	0·49	0·49	0·60	0·61	0·55	0·55
Lower middle school	1·4 ^b^	1·4 ^b^	4·9 ^b^	4·9 ^b^	1·2 ^b^	1·2 ^b^
P	0·91	0·91	0·09	0·09	0·24	0·24
Upper middle school	−1·9 ^a^	−1·9 ^a^	4·3 ^b^	4·3 ^b^	3·1 ^b^	3·1 ^b^
P	0·22	0·22	0·21	0·21	0·07	0·07
Vocational degree	−5·5 ^a^	−5·5 ^a^	−1·3 ^b^	−1·3 ^b^	−1·8 ^c^	−1·8 ^c^
P	<0·01	<0·01	0·73	0·74	0·91	0·91
University degree or higher	−6·1 ^a^	−6·1 ^a^	5·5 ^b^	5·5 ^b^	3·9 ^b^	3·9 ^b^
P	<0·01	<0·01	0·21	0·21	0·14	0·15
Income ^3^	1·0 ^a^	1·0 ^a^	1·5 ^b^	1·5 ^b^	−1·9 ^c^	−1·9 ^c^
P	<0·01	0·01	0·13	0·13	0·67	0·68
Sedentary ^4^	−7·4 ^c^	−7·4 ^c^	2·2 ^c^	2·2 ^c^	1·6 ^c^	1·6 ^c^
P	0·60	0·60	0·55	0·54	0·22	0·22
Exercise ^5^	6·6 ^b^	6·6 ^b^	−2·5 ^b^	−2·5 ^b^	4·8 ^c^	4·8 ^c^
P	0·28	0·29	0·09	0·09	0·30	0·34
Labour intensity ^6^						
Moderate	−8·9 ^b^	−8·9 ^b^	−6·0 ^b^	−6·0 ^b^	−2·7 ^b^	−2·7 ^b^
P	0·34	0·34	0·04	0·04	0·02	0·02
Heavy	−6·8 ^a^	−6·8 ^a^	−1·2 ^a^	−1·2 ^a^	−1·5 ^b^	−1·5 ^b^
P	<0·01	<0·01	<0·01	<0·01	0·22	0·22
Drinking ^7^						
Very low	−3·0 ^b^	−3·0 ^b^	−5·8 ^b^	−5·8 ^b^	−2·3 ^b^	−2·3 ^b^
P	0·83	0·83	0·10	0·09	0·13	0·13
Low	3·6 ^a^	3·6 ^a^	−6·8 ^b^	−6·8 ^b^	2·6 ^c^	2·6 ^c^
P	<0·01	<0·01	0·03	0·03	0·88	0·88
Medium	7·7 ^a^	7·7 ^a^	−1·4 ^b^	−1·4 ^b^	−2·4 ^b^	−2·4 ^b^
P	<0·01	<0·01	0·69	0·69	0·06	0·06
High	8·6 ^a^	8·6 ^a^	−9·3 ^b^	−9·3 ^b^	−3·9 ^b^	−3·9 ^b^
P	<0·01	<0·01	0·01	0·01	<0·01	<0·01
Very high	6·4 ^b^	6·4 ^b^	−1·1 ^a^	−1·1 ^a^	−2·5 ^b^	−2·5 ^b^
P	0·63	0·63	<0·01	<0·01	0·02	0·02
Smoking	−3·1 ^b^	−3·1 ^b^	−3·2 ^c^	−3·2 ^c^	−9·4 ^d^	−9·4 ^d^
P	<0·01	<0·01	0·02	0·02	0·17	0·17
Household size	−1·2 ^a^	−1·2 ^a^	−1·4 ^b^	−1·4 ^b^	−3·1 ^c^	−3·1 ^c^
P	<0·01	<0·01	0·03	0·03	0·28	0·28
Urbanisation ^8^	5·8 ^c^	5·8 ^c^	2·9 ^c^	2·9 ^c^	−1·3 ^d^	−1·3 ^d^
P	0·02	0·03	<0·01	<0·01	0·66	0·66
2006	7·1 ^b^	7·1 ^b^	3·8 ^b^	3·8 ^b^	8·5 ^c^	8·5 ^c^
P	0·23	0·25	0·14	0·14	0·48	0·48
2009	2·7 ^a^	2·7 ^a^	1·1 ^a^	1·1 ^a^	2·7 ^b^	2·7 ^b^
P	<0·01	<0·01	<0·01	<0·01	0·02	0·02
2011	5·7 ^a^	5·7 ^a^	1·3 ^a^	1·3 ^a^	1·4 ^c^	1·4 ^c^
P	<0·01	<0·01	<0·01	<0·01	0·90	0·90
Wald *χ* ^2^(23)	904·12	852·82	699·7	690·71	210·06	210·13
*P* value > *χ* ^2^	<0·01	<0·01	<0·01	<0·01	<0·01	<0·01
Pseudo *R*-square	0·03	0·03	0·15	0·15	0·13	0·13
Correctly classified (%)	61·1	61·1	98·1	98·1	99·6	99·6

OW, DM and MI are acronyms of overweight, diabetes mellitus and myocardial infarction, respectively. Overweight is defined as BMI ≥ 24 kg/m^2^. Ind denotes individual cluster effect; Hh denotes household cluster effect. ^1^ Gender: 1 = male, 0 = female; ^2^ Highest education completed: 1 = no school completed (reference group), 2 = primary school, 3 = lower middle school, 4 = upper middle school, 5 = vocational degree, 6 = undergraduate or higher degrees; ^3^ The annual household net income per capita after deflation at 2015 prices and then taking natural logarithm; ^4^ Daily sedentary activity time (hours); ^5^ Daily physical exercise time (minutes); ^6^ Labour intensity levels: 1 = light physical activity (reference category), working in a sitting or standing position (e.g. office work, counter salesperson); 2 = moderate physical activity (e.g. driver, electrician); 3 = heavy physical activity (e.g. farmer, athlete, steel worker, lumber worker); ^7^ The frequency of drinking alcohol, 1 = no drinking (reference group); 2 = very low frequency, no more than once a month; 3 = low frequency, once or twice a month; 4 = medium frequency, once or twice a week; 5 = high frequency, 3–4 times a week; 6 = very high frequency, almost every day; ^8^ Defined by a multidimensional 12-component urbanisation index, including the population density, physical, social, cultural, and economic environment. ^a^ The coefficient is displayed in scientific notation format: coefficient × 10^−2^; ^b^ scientific notation format: coefficient × 10^−3^; ^c^ scientific notation format: coefficient × 10^−4^; ^d^ scientific notation format: coefficient × 10^−5^.

**Table A5:** The associations between diet quality indices and daily energy intake from OLS using individual cluster effect (*n* 30 350)

	(1)	(2)	(3)	(4)	(5)	(6)	(7)
Count	21·1						
P	<0·01						
DDS		133·4					
P		<0·01					
BI			617·4				
P			<0·01				
EI				231·6			
P				<0·01			
CHDI					−14·2		
P					<0·01		
CFPS						−17·6	
P						<0·01	
DQD							89·2
P							<0·01
Gender ^1^	298·8	304·3	297·7	302·3	286·3	288·0	256·2
P	<0·01	<0·01	<0·01	<0·01	<0·01	<0·01	<0·01
Age	−0·9	−1·0	−1·0	−0·9	−0·9	−1·0	−0·8
P	0·03	0·01	0·01	0·02	0·03	0·02	0·04
Education ^2^							
Primary school	−38·1	−48·0	−35·4	−39·2	−31·1	−30·0	−21·0
P	0·01	<0·01	0·01	<0·01	0·02	0·03	0·10
Lower middle school	−23·8	−33·6	−17·2	−24·8	−8·6	−8·6	−3·5
P	0·08	0·01	0·21	0·07	0·52	0·53	0·78
Upper middle school	−36·3	−45·0	−26·1	−36·5	−18·1	−15·5	−8·7
P	0·03	0·01	0·12	0·03	0·28	0·36	0·58
Vocational degree	−46·3	−59·8	−26·8	−41·0	−21·0	−16·7	−19·7
P	0·02	<0·01	0·17	0·04	0·29	0·40	0·29
University degree or higher	−142·3	−148·4	−107·2	−126·9	−105·3	−97·0	−75·0
P	<0·01	<0·01	<0·01	<0·01	<0·01	<0·01	<0·01
Income ^3^	32·0	31·2	44·0	35·5	53·9	51·4	44·8
P	<0·01	<0·01	<0·01	<0·01	<0·01	<0·01	<0·01
Sedentary ^4^	−5·0	−4·9	−2·9	−4·0	−2·3	−2·0	−2·2
P	<0·01	<0·01	0·09	0·02	0·19	0·25	0·18
Exercise ^5^	7·0	6·4	15·3	11·6	18·2	17·5	14·3
P	0·35	0·38	0·04	0·11	0·01	0·02	0·04
Labo u r intensity ^6^							
Moderate	76·1	76·5	69·8	75·2	67·6	66·7	62·1
P	<0·01	<0·01	<0·01	<0·01	<0·01	<0·01	<0·01
Heavy	221·0	213·4	209·2	222·1	194·5	195·9	185·4
P	<0·01	<0·01	<0·01	<0·01	<0·01	<0·01	<0·01
Drinking ^7^							
Very low	−27·8	−18·5	−11·2	−16·8	−9·6	−11·1	−8·7
P	0·13	0·31	0·55	0·36	0·60	0·55	0·62
Low	40·3	43·9	52·1	47·0	53·9	53·6	42·8
P	0·01	<0·01	<0·01	<0·01	<0·01	<0·01	<0·01
Medium	32·1	39·7	47·5	38·8	54·4	52·2	41·9
P	0·03	0·01	<0·01	0·01	<0·01	<0·01	<0·01
High	91·9	101·8	109·3	99·8	116·7	114·6	89·9
P	<0·01	<0·01	<0·01	<0·01	<0·01	<0·01	<0·01
Very high	155·8	172·1	176·0	165·1	183·8	183·4	147·9
P	<0·01	<0·01	<0·01	<0·01	<0·01	<0·01	<0·01
Smoking	1·6	1·6	1·4	1·5	1·5	1·5	1·1
P	<0·01	<0·01	0·01	0·01	0·01	0·01	0·04
Household size	−16·4	−8·7	−10·2	−11·5	−9·0	−11·9	−7·1
P	<0·01	<0·01	<0·01	<0·01	<0·01	<0·01	0·01
Urbanisation ^8^	−3·1	−2·9	−1·8	−2·8	−0·7	−1·0	−1·1
P	<0·01	<0·01	<0·01	<0·01	0·02	<0·01	<0·01
2006	−30·4	−33·1	−27·2	−34·5	−10·8	−18·6	−23·1
P	<0·01	<0·01	0·01	<0·01	0·27	0·06	0·01
2009	−96·9	−95·1	−88·3	−101·4	−64·4	−73·9	−73·3
P	<0·01	<0·01	<0·01	<0·01	<0·01	<0·01	<0·01
2011	−337·8	−314·9	−296·7	−324·9	−265·4	−277·6	−250·8
P	<0·01	<0·01	<0·01	<0·01	<0·01	<0·01	<0·01
Constant	1778·1	1515·4	1319·5	1489·6	2441·4	1741·5	1292·7
P	<0·01	<0·01	<0·01	<0·01	<0·01	<0·01	<0·01
*F*(23, 14 727)	209·06	211·66	178·67	196·75	192·73	174·21	253·97
*P* value > *F*	<0·01	<0·01	<0·01	<0·01	<0·01	<0·01	<0·01
*R*-squared	0·17	0·17	0·15	0·16	0·16	0·15	0·23

^1^ Gender: 1 = male, 0 = female; ^2^ Highest education completed: 1 = no school completed (reference group), 2 = primary school, 3 = lower middle school, 4 = upper middle school, 5 = vocational degree, 6 = undergraduate or higher degrees; ^3^ The annual household net income per capita after deflation at 2015 prices and then taking natural logarithm; ^4^ Daily sedentary activity time (hours); ^5^ Daily physical exercise time (minutes); ^6^ Labour intensity levels: 1 = light physical activity (reference category), working in a sitting or standing position (e.g. office work, counter salesperson); 2 = moderate physical activity (e.g. driver, electrician); 3 = heavy physical activity (e.g. farmer, athlete, steel worker, lumber worker); ^7^ The frequency of drinking alcohol, 1 = no drinking (reference group); 2 = very low frequency, no more than once a month; 3 = low frequency, once or twice a month; 4 = medium frequency, once or twice a week; 5 = high frequency, 3–4 times a week; 6 = very high frequency, almost every day; ^8^ Defined by a multidimensional 12-component urbanisation index, including the population density, physical, social, cultural, and economic environment.

**Table A6:** The associations between diet quality indices and daily energy intake from OLS using household cluster effect (*n* 30 350)

	(1)	(2)	(3)	(4)	(5)	(6)	(7)
Count	21·1						
P	<0·01						
DDS		133·4					
P		<0·01					
BI			617·4				
P			<0·01				
EI				231·6			
P				<0·01			
CHDI					−14·2		
P					<0·01		
CFPS						−17·6	
P						<0·01	
DQD							89·2
P							<0·01
Gender ^1^	298·8	304·3	297·7	302·3	286·3	288·0	256·2
P	<0·01	<0·01	<0·01	<0·01	<0·01	<0·01	<0·01
Age	−0·9	−1·0	−1·0	−0·9	−0·9	−1·0	−0·8
P	0·04	0·01	0·02	0·03	0·04	0·02	0·06
Education ^2^							
Primary school	−38·1	−48·0	−35·4	−39·2	−31·1	−30·0	−21·0
P	0·01	<0·01	0·02	0·01	0·04	0·04	0·13
Lower middle school	−23·8	−33·6	−17·2	−24·8	−8·6	−8·6	−3·5
P	0·10	0·02	0·24	0·09	0·56	0·56	0·80
Upper middle school	−36·3	−45·0	−26·1	−36·5	−18·1	−15·5	−8·7
P	0·05	0·01	0·15	0·05	0·32	0·40	0·61
Vocational degree	−46·3	−59·8	−26·8	−41·0	−21·0	−16·7	−19·7
P	0·03	0·01	0·22	0·06	0·33	0·44	0·33
University degree or higher	−142·3	−148·4	−107·2	−126·9	−105·3	−97·0	−75·0
P	<0·01	<0·01	<0·01	<0·01	<0·01	<0·01	<0·01
Income ^3^	32·0	31·2	44·0	35·5	53·9	51·4	44·8
P	<0·01	<0·01	<0·01	<0·01	<0·01	<0·01	<0·01
Sedentary ^4^	−5·0	−4·9	−2·9	−4·0	−2·3	−2·0	−2·2
P	0·01	0·01	0·13	0·04	0·25	0·31	0·23
Exercise ^5^	7·0	6·4	15·3	11·6	18·2	17·5	14·3
P	0·38	0·41	0·05	0·14	0·02	0·03	0·06
Labor intensity ^6^							
Moderate	76·1	76·5	69·8	75·2	67·6	66·7	62·1
P	<0·01	<0·01	<0·01	<0·01	<0·01	<0·01	<0·01
Heavy	221·0	213·4	209·2	222·1	194·5	195·9	185·4
P	<0·01	<0·01	<0·01	<0·01	<0·01	<0·01	<0·01
Drinking ^7^							
Very low	−27·8	−18·5	−11·2	−16·8	−9·6	−11·1	−8·7
P	0·14	0·33	0·56	0·38	0·61	0·56	0·62
Low	40·3	43·9	52·1	47·0	53·9	53·6	42·8
P	0·01	0·01	<0·01	<0·01	<0·01	<0·01	<0·01
Medium	32·1	39·7	47·5	38·8	54·4	52·2	41·9
P	0·03	0·01	<0·01	0·01	<0·01	<0·01	<0·01
High	91·9	101·8	109·3	99·8	116·7	114·6	89·9
P	<0·01	<0·01	<0·01	<0·01	<0·01	<0·01	<0·01
Very high	155·8	172·1	176·0	165·1	183·8	183·4	147·9
P	<0·01	<0·01	<0·01	<0·01	<0·01	<0·01	<0·01
Smoking	1·6	1·6	1·4	1·5	1·5	1·5	1·1
P	0·01	<0·01	0·01	0·01	0·01	0·01	0·05
Household size	−16·4	−8·7	−10·2	−11·5	−9·0	−11·9	−7·1
P	<0·01	0·04	0·02	0·01	0·04	0·01	0·07
Urbanisation ^8^	−3·1	−2·9	−1·8	−2·8	−0·7	−1·0	−1·1
P	<0·01	<0·01	<0·01	<0·01	0·09	0·01	<0·01
2006	−30·4	−33·1	−27·2	−34·5	−10·8	−18·6	−23·1
P	0·02	0·01	0·04	0·01	0·41	0·16	0·06
2009	−96·9	−95·1	−88·3	−101·4	−64·4	−73·9	−73·3
P	<0·01	<0·01	<0·01	<0·01	<0·01	<0·01	<0·01
2011	−337·8	−314·9	−296·7	−324·9	−265·4	−277·6	−250·8
P	<0·01	<0·01	<0·01	<0·01	<0·01	<0·01	<0·01
Constant	1778·1	1515·4	1319·5	1489·6	2441·4	1741·5	1292·7
P	<0·01	<0·01	<0·01	<0·01	<0·01	<0·01	<0·01
*F*(23, 14 727)	266·83	269·80	244·06	258·28	255·13	239·59	309·01
*P* value > *F*	<0·01	<0·01	<0·01	<0·01	<0·01	<0·01	<0·01
*R*-squared	0·17	0·17	0·15	0·16	0·16	0·15	0·23

^1^ Gender: 1 = male, 0 = female; ^2^ Highest education completed: 1 = no school completed (reference group), 2 = primary school, 3 = lower middle school, 4 = upper middle school, 5 = vocational degree, 6 = undergraduate or higher degrees; ^3^ The annual household net income per capita after deflation at 2015 prices and then taking natural logarithm; ^4^ Daily sedentary activity time (hours); ^5^ Daily physical exercise time (minutes); ^6^ Labour intensity levels: 1 = light physical activity (reference category), working in a sitting or standing position (e.g. office work, counter salesperson); 2 = moderate physical activity (e.g. driver, electrician); 3 = heavy physical activity (e.g. farmer, athlete, steel worker, lumber worker); ^7^ The frequency of drinking alcohol, 1 = no drinking (reference group); 2 = very low frequency, no more than once a month; 3 = low frequency, once or twice a month; 4 = medium frequency, once or twice a week; 5 = high frequency, 3–4 times a week; 6 = very high frequency, almost every day; ^8^ Defined by a multidimensional 12-component urbanisation index, including the population density, physical, social, cultural, and economic environment.
